# Integrating morphological and phytochemical studies on some selected taxa of Lamiaceae Lindl. and Verbenaceae Juss.

**DOI:** 10.1186/s12870-026-08910-2

**Published:** 2026-05-20

**Authors:** Jackline S. Garas, Mohamed A. Salim, Mariam I. Hussein, Magdy M. Mourad, Nagwa M. Ammar, Mohamed M. Ayoub

**Affiliations:** 1 Department of Botany, Faculty of Science, Ain Shams University, Cairo, Egypt; 2 Mazhar Botanic Garden, Giza, Egypt; 3 Department of Pharmacognosy, Pharmaceutical and Drug Industries Research Institute, National Research Centre, Giza, Egypt

**Keywords:** Cytotoxicity, Essential oil, GC-MS, HepG 2, Lamiaceae, Morphology, Verbenaceae

## Abstract

**Background:**

Lamiaceae and Verbenaceae are taxonomically sister families, belong to the order Lamiales. They are well known for their phytochemical constituents with bio-active properties, but considerable taxonomic difficulties in discriminating between the two families have been reported. The present study aims to explore the morpho-anatomical characters (stem and lamina) of 11 selected species bearing essential oils (EOs) by using the light microscope (LM), and to identify EO constituents in their aerial parts using gas chromatography–mass spectrometry (GC-MS), for taxonomic delimitation among these species. The essential oils of the species with the highest EO yield were tested in-vitro against a human hepatocellular carcinoma (HepG 2) cell line.

**Results:**

The macro- and micromorphological characters of the stem and lamina were recorded. From these characters trichomes, ground system, and vascular bundles were diagnostic at the species level. GC-MS analysis detected the existence of 160 constituents prevailed by monoterpenes and sesquiterpenes classes in all species under investigation except in *Citharexylum spinosum* L. A, monoterpenes (2.19%) and sesquiterpenes (0.3%). In-vitro bioassay of EO against HepG 2 revealed a significant effect, as evidenced by the IC_50_ values being 58.9, 48.3, 36.7 and 34.5 µg/mL for *Mentha spicata* L., *Salvia microphylla* Kunth., *Lantana camara* L. and *Ocimum labiatum* (N.E.Br.) A.J. Paton respectively.

**Conclusions:**

This work revealed that Lamiaceae and Verbenaceae families are closely related, showing fluctuation in the mutual morpho-anatomical characteristics of the studied species, however, EO analysis showed variation among these species.

**Supplementary Information:**

The online version contains supplementary material available at 10.1186/s12870-026-08910-2.

## Introduction

Lamiaceae Lindl. (mint family) and Verbenaceae Juss. (verbena family) are the two core families of order Lamiales. Lamiaceae is the sixth largest angiosperm family and represents the largest family-level clade within Lamiales. This family contains about 236 genera and 6900–7200 species, and is subdivided into 12 subfamilies considered cosmopolitan mainly in tropical and subtropical regions but absent from coldest regions of high latitude or altitude [1–5]. Whereas, Verbenaceae has approximately 32 genera and 800 species, mostly distributed in tropical and subtropical Americas [6]. The economic relevance of the Lamiaceae family is related not only to the presence of the essential oil rich in active compounds, but also to its medicinal uses, ornamental, perfumes, cosmetics and culinary advantages [1, 7, 8]. The Verbenaceae is well known for constituents having important bio-active properties as well [9].

Lamiaceae has been considered closely related to Verbenaceae; in the 1990s, phylogenetic studies suggested that many genera classified in Verbenaceae should be transferred to Lamiaceae or other families under the order Lamiales. Several important timber plants formerly placed in Verbenaceae have been reassigned to Lamiaceae according to APG II & III, such as *Callicarpa americana* L., *Premna odorata* Blanco., *Tectona grandis* L.f. and *Vitex angus-castus* L., so the phylogenetic backbone has never been resolved, and several long-standing problems regarding the boundaries between the two families have arisen [10, 11].

Morpho-anatomically, many species in both families share some characteristics such as the bisexual bilabial flowers gathered in inflorescences, opposite decussate leaves with symmetrical bases, and the dorsiventral lamina that is discriminated into palisade and spongy layers [1, 12, 13]. Many authors highlighted that glandular and eglandular trichomes are frequently present in many species of both Lamiaceae and Verbenaceae [14–18].

Most Lamiaceae and Verbenaceae plants are characteristically aromatic due to their contents of EO which is a mixture of odorous volatile compounds in form of secondary metabolites, particularly, of terpenoid nature, normally formed in special cells like bulbous secretory glands found in leaves and stems, and when they occur in various organs in the same plant, they frequently have different composition profiles [9]. Such volatile products accumulate with very diverse structures and many of them are reported as chemotaxonomic markers at all taxonomic levels [19, 20]. These products evaporate quickly when exposed to the air at ordinary temperature, play a role in the defense system of higher plants, and are used to give flavor to foods and drinks and as fragrances, as monoterpenes and sesquiterpenes are particularly responsible for the characteristic flavor and fragrance of such plants [21–23].

Biologically, recent studies revealed that the chemical composition of many Lamiaceae and Verbenaceae members possesses great medicinal potential represented by their antimicrobial, antioxidant, cytotoxic, anti-inflammatory and hepatoprotective activities due to flavonoid, iridoid and terpenoid constituents [8, 19, 24].

The aim of this work is to ensure the discriminations between both families and the characterization of each family member in this study by investigating the diversity of macromorphological characters of the whole plant and micromorphological characters (stem and lamina) using light microscopy, as well as identification of their essential oils’ constituents by GC-MS analysis and evaluation of their cytotoxic activity against hepatocellular carcinoma HepG 2 cell line. Thus, the present work is an attempt to find possible correlations between the morpho-anatomical features and the phytochemical composition of the studied species of Lamiaceae and Verbenaceae selected for containing essential oil.

## Materials and methods

### Plant samples

In the present study, fresh specimens of 11 species; eight species belonging to the Lamiaceae family and three species of the Verbenaceae family, were collected during the spring and early summer of 2024 in the flowering stage from different localities in Egypt. The authors identified the plants with the aid of [12, 25–27]. Updated nomenclature and synonyms were derived from Plants of the World Online (POWO) [28]. The voucher specimens were deposited in the Herbarium of Mazhar Botanic Garden (MAZHAR). The synonyms, localities, and voucher numbers of the studied species are shown in Table 1.

**Table 1:** Collection data of the studied species

No.	Species	Family	Locality	Voucher No.
**1**	***Callicarpa americana*** L.	Lamiaceae	**A**	EGY-MAZHAR011304
2	***Mentha spicata*** L. = *M. microphylla* K.Koch		//	EGY-MAZHAR022301
3	***Ocimum labiatum*** (N.E.Br.) A.J.Paton = *Orthosiphon labiatus* N.E.Br.		//	EGY-MAZHAR030102
4	***Premna odorata*** Blanco.		//	EGY-MAZHAR030801
5	***Salvia microphylla*** Kunth.		//	EGY-MAZHAR031402
6	***Tectona grandis*** L.f. *= T. theca* Lour. *= Theka grandis* (L.f.) Lam.		//	EGY-MAZHAR032203
7	***Thymus vulgaris*** L.		//	EGY-MAZHAR032303
8	***Vitex agnus-castus*** L		//	EGY-MAZHAR032505
9	***Aloysia gratissima*** (Gillies & Hook.) Tronc. = *Verbena gratissima* Gillies & Hook.	Verbenaceae	//	EGY-MAZHAR010502
10	***Citharexylum spinosum*** L. A *= C. quadrangulare* Jacq.		B	EGY-MAZHAR011803
11	***Lantana camara*** L. *= Camara vulgaris* Benth. *= L. undulata* Raf.		C	EGY-MAZHAR021801

A= Mazhar Botanic Garden, Giza, Egypt. (30° 03' N, 31° 08' E)

B= Qubba Palace, Cairo, Egypt. (31° 32' N, 31° 31' E)

C= Campus of Ain Shams University. (30° 08' N, 31° 28' E)

### Morphological studies

The macromorphological characters of the whole plant, such as habit, stem, leaf, inflorescence and flower were examined and described directly from fresh samples (five individuals per species at least). The laminar sizes were measured to determine the leaf classes according to the Leaf Architecture Working Group (Manual of leaf architecture) [29]. Different leaf classes were obtained as shown in Table 2.

**Table 2:** Macromorphological characters of the studied species (character states and their codes)

Characters & Character States with their Codes				Species										
				1	2	3	4	5	6	7	8	9	10	11
Habit: tree (1), shrub **(2)**, subshrub **(3)**, herb **(4)**				3	4	3	1	4	1	4	2	2	1	3
Stem texture: pubescent **(1)**, tomentose **(2)**, glabrous **(3)**				2	2	2	2	1	1	1	3	1	3	2
Leaf	**Duration**: deciduous **(1)**, evergreen to semideciduous **(2)**, evergreen **(3)**			1	2	2	3	3	2	3	2	2	3	2
	**Composition**: simple **(1)**, compound **(2)**			1	1	1	1	1	1	1	2	1	1	1
	**Insertion**: petiolate **(1)**, sessile **(2)**			1	2	1	1	1	1	1	1	1	1	1
	Lamina	**Laminar size class**: leptophyll **(1)**, nanophyll **(2)**, microphyll **(3)**, notophyll **(4)**, mesophyll **(5)**, macrophyll **(6)**		4	3	3	5	3	6	1	5	2	5	3
		**Shape**: ovate **(1)**, lanceolate **(2)**, oblong - elliptic **(3)**, ovate - elliptic **(4)**, cordate **(5)**		1	1	5	5	4	4	3	2	4	3	1
		**Margin**: serrate **(1)**, dentate **(2)**, entire **(3)**, undulate **(4)**		2	1	2	4	2	4	3	3	3	4	2
		**Apex**: acuminate **(1)**, acute **(2)**		1	2	2	1	2	1	2	1	2	1	1
		**Base shape**: truncate to cordate **(1)**, cuneate **(2)**, acute **(3)**		2	1	1	1	3	2	3	2	3	2	3
		**Texture**: pubescent **(1)**, scabrous **(2)**, tomentose **(3)**		2	3	1	3	1	2	1	1	1	1	2
Flower	**Inflorescence**: simple raceme **(1)**, cymes in terminal panicles **(2)**, compressed racemose **(3)**, axillary cyme **(4)**, verticillaster **(5)**, spike **(6)**			4	5	1	2	1	2	4	2	6	1	3
	**Floral parts**: tetramerous **(1)**, pentamerous **(2)**, tetra & pentamerous **(3)**			2	3	3	3	3	2	3	3	1	3	2
	**Symmetry**: actinomorphic **(1)**, zygomorphic **(2)**			1	2	2	2	2	1	2	2	1	2	2
	**Color**: white **(1)**, pink **(2)**, purple **(3)**, mixed **(4)**			2	3	3	1	2	1	1	3	1	1	4
	**Stamens number**: 2 **(1)**, 4 **(2)**, 4 to 6 **(3)**			3	2	2	2	1	3	2	2	2	2	2
*Fruit	**Type**: drupe **(1)**, schizocarp **(2)**			1	2	2	1	2	1	2	1	2	1	1
	**Shape**: globose **(1)**, elliptic **(2)**			1	2	2	1	2	1	2	1	2	1	1
	**Color**: brownish **(1)**, reddish orange **(2)**, purple **(3)**, green to black **(4)**			3	1	1	4	1	1	1	1	1	2	4
	**Seed shape**: nutlets **(1)**, ovoid to globose **(2)**, flattened ellipsoid **(3)**			3	1	1	2	1	2	1	2	1	2	2

(*) refers to that some data about the fruits of the studied taxa 2, 3, 5, 7 and 9 have not been examined in this study but taken from other sources [25, 26]

Stem and lamina micromorphological features were examined through thin transverse sections cut by hand microtome at 13–16 μm (between the fourth and fifth node for the stem and at the middle portion of the lamina), then double stained by safranine and light green combination and fixed using Canada Balsam according to the traditional method of Johansen [30]. The sections were examined using BEL: B103 T-PL light microscope and photographed using Digital Camera (Canon power-shot A720, 8 megapixels) at the laboratory of Taxonomy Unit, Department of Botany, Faculty of Science, Ain Shams University. The terminology for micromorphological study follows Metcalfe and Chalk [27].

### Essential oil preparation

The essential oils were prepared from the collected plant samples (300–450 g of aerial parts), by the hydrodistillation extraction technique, using a Clevenger-type apparatus, in a laboratory at the Department of Pharmacognosy, Pharmaceutical and Drug Industries Institute, National Research Centre. Fresh leaves were manually comminuted using scissors and placed in a 5 L round-bottom flask containing distilled water. The mixture was heated to boiling, and hydrodistillation was maintained for 4 h. Following extraction, the essential oils (EOs) were separated from the aqueous phase and dried over anhydrous sodium sulfate. The resulting pure EOs were stored in amber glass vials at ambient temperature in a refrigerator and sheltered from light. Yields were calculated in volume/mass percentage (v/w%) of dry weight (the essential oil yield was calculated as a percentage relative to the initial weight of each sample to ensure comparability), i.e. volume (mL) of essential oil per mass (g) of plant material [31].

### Essential oil analysis

For analysis of the previously extracted EOs, mass spectra were conducted using Shimadzu GCMS-QP2010 (Kyoto, Japan) coupled with Rtx-5MS fused bonded column (30 m x 0.25 mm i.d. x 0.25 μm film thickness) (Restek, USA) equipped with a split–splitless injector. The initial column temperature was retained at 45 °C for 2 min (isothermal) and programmed to 300 °C at a rate of 5 °C/min and kept constant at 300 °C for 5 min (isothermal). Injector temperature was 250 °C. The carrier gas was Helium at a flow rate of 1.41 mL/min. All the mass spectra were set at the following condition: (equipment current) filament emission current, 60 mA; ionization voltage, 70 eV; ion source, 200 °C. Diluted samples (1% v/v) were injected with split mode (split ratio 1: 15) [32]. Identification of the volatile constituents was attained by comparing their retention indices (RI) with those of standard n-alkane series (C_8_–C_28_) and their mass spectra with those reported in the NIST (National Institute of Standards and Technology) database, ensuring that the similarity index (SI) > 85% [33].

### *In-vitro* Cytotoxic Activity

In vitro bioassay on human tumor (hepatocellular carcinoma) cell lines (HepG 2) conducted by the Bioassay-Cell Culture Laboratory, National Research Centre, Giza, Egypt was used in cell viability assessment by the mitochondrial-dependent reduction of yellow MTT (3-[4,5-dimethylthiazole-2-yl]-2,5-diphenyl tetrazolium bromide) into purple formazan [34]. The method was done according to Thabrew et al. and El-Menshawi et al. [35, 36], in a sterile area using a laminar flow cabinet biosafety class II level (Baker, SG403INT, Sanford, ME, USA). Cytotoxic activity of essential oils extracted from the aerial parts of the studied taxa was tested using the cell line technique according to Cordero et al. [37]. Doxorubicin was used as a standard reference drug (positive control) The absorbance was measured by a microplate multi-well reader (Bio-Rad Laboratories Inc., model 3350, Hercules, California, USA) at 595 nm and a reference wavelength 620 nm. Statistical significance was tested between samples and negative control (cell with vehicle) using an independent T-test by SPSS 11 program. Dimethyl sulfoxide (DMSO) was the vehicle used for dissolution of plant extracts and its concentration on the cells was less than 0.2%. The percentage of change of viability was calculated according to the formula: [(Absorbance of tested extract/ Absorbance of negative control)^−1^] x100.

A probit analysis was carried out for IC_50_ determination using SPSS 11 program. In the present study, the IC₅₀ values were calculated to compare the anticancer potency of the tested samples, where IC_50_ is the concentration required to kill 50% of the cell population.

## Results

### Morphological characters

#### Macromorphological investigation

The selected plants for this study display diverse macromorphological characters, as shown in Table 2, including 20 character states that were extracted directly from the fresh specimens, indicating that Lamiaceae members have different growth habits; trees, shrubs, subshrub or herbs developing woody to herbaceous stems. Flowers are either radially symmetrical (actinomorphic) in *Callicarpa americana* and *Tectona grandis*, or bilaterally symmetrical (zygomorphic) in the remaining species, and later form schizocarpic fruits with four nutlets or drupes. The studied taxa of Verbenaceae are trees, shrubs, or subshrubs, and bear flowers in panicles that vary in color and form (raceme or spike). All studied taxa in both Lamiaceae and Verbenaceae are perennials, monoecious, with opposite decussate and symmetric lamina base. Leaves are mostly simple pinnate, except in *Vitex agnus-castus*, which is compound palmate. The lamina size, shape, margin, apex and base shapes showed great variation among all the studied species. The flowers are bisexual and arranged in panicles or verticillasters of variable forms.

#### Micromorphological investigation

##### Stem anatomical characteristics (Fig. 1; Table 3)

The outline of stem sections in Lamiaceae is terete in *Premna odorata* and *Vitex agnus-castus*, more or less rounded in *Callicarpa americana* and *Thymus vulgaris*, and square in the others (deeply ridged and furrowed in *Tectona grandis*). Regarding Verbenaceae, it is also square in *Citharexylum spinosum*,* Lantana camara*, and terete in *Aloysia gratissima*. Trichomes varied in type and can be categorized as; (a) eglandular trichomes including two subtypes; (1) acicular hairs with high density, differ in being uni-, bi-, and multicellular, but completely absent in *C. spinosum*, (2) candelabra-like trichomes were detected only in *C. americana* and *T. grandis*, (b) glandular trichomes with low density, including the labiaceous subtype of peltate glandular structures (Fig. 2a), that is seldom in *Salvia microphylla*, *T. vulgaris*, *A. gratissima*, *C. americana* and *T. grandis*, but abundant in *Mentha spicata*.

**Fig. 1:**
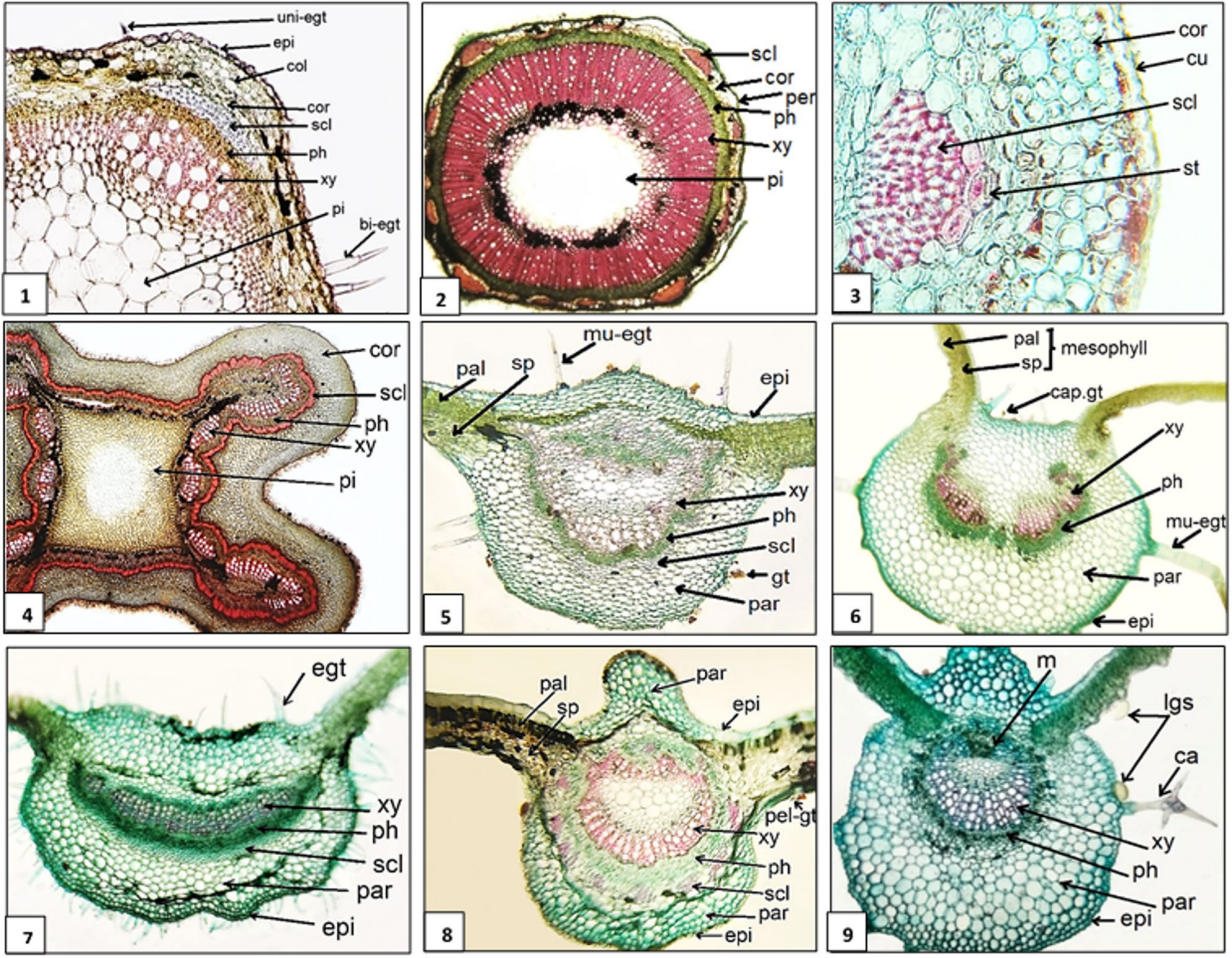
Major Aspects of Micromorphological Characters of the Stem and Lamina by LM: Text Figs. 1–4: Major aspects of stem anatomical characters: (**1**) square outline and solid pith of polyhedral parenchyma in *Salvia microphylla*; (**2**) terete outline, hollow pith, and continuous cylinder of vascular system in *Aloysia gratissima*; (**3**) stone cells in *Citharexylum spinosum*; (**4**) complete ring of extraxylary fibers, and square outline with lobed angles in *Tectona grandis*. Text Figs. **5-9**: Major aspects of lamina anatomical characters: (**5**) dorsiventral mesophyll with connected palisade layer in *Premna odorata*; (**6**) dissected vascular system in *Ocimum labiatum*; (**7**) crescent-shaped vascular system in *Mentha spicata*; (**8**) patches of sclerenchyma, and a cylinder of vascular system in *C. spinosum*; (**9**) horseshoe-shaped vascular system, and one accessory bundle in *Callicarpa americana*. bi-egt: bicellular eglandular trichome, ca.: candelabra trichome, cap-gt: capitate glandular trichome (stalked), col: collenchyma, cor: cortical parenchyma (cortex), cu: cuticle, egt: eglandular trichome, epi: epidermis, gt: glandular trichome, lgs: labiaceous glandular structure, m: median (accessory) bundle, mu-egt: multicellular eglandular trichome, pal: palisade, par: parenchyma, pel-gt: peltate glandular trichome, per: periderm, ph: phloem, pi: pith, scl: sclerenchyma, sp: spongy, st: stone cells, xy: xylem

**Table 3:** Stem anatomical characteristics of the studied species (character states and their codes)

Characters & Character States with their Codes						Species										
						1	2	3	4	5	6	7	8	9	10	11
Outline: terete (1), square (2), ± rounded (3)						3	2	2	1	2	2	3	1	1	2	2
Dermal tissue	**Epidermis**: tangential **(1)**, radial **(2)**, radial to tangential **(3)**, radial at corners & tangential elsewhere **(4)**					1	1	4	2	4	2	1	2	4	1	3
	**Hypodermis**: absent **(1)**, present **(2)**					1	2	1	1	1	1	1	1	1	1	2
	**Cuticle thickness**: thin **(1)**, thick **(2)**					2	2	1	2	1	2	2	2	2	2	2
	**Trichomes**	**Glandular**	**Capitate**	**Stalked**: absent **(1)**, present **(2)**		1	1	2	2	2	1	1	2	2	1	2
				**Sessile**: absent (1), present (2)		1	2	1	1	2	2	2	1	2	1	2
			**Peltate**:	**Shield-shaped**: absent **(1)**, present **(2)**		2	1	1	1	1	1	2	1	1	2	2
				**Multicellular-head**: absent **(1)**, present **(2)**		1	1	2	2	1	1	1	2	1	1	2
				**Labiaceous**: absent **(1)**, seldom **(2)**, abundant **(3)**		3	2	1	1	2	2	2	1	2	1	1
		**Eglandular**	**Unbranched uniseriate unicellular**: absent **(1)**, present **(2)**			2	1	1	1	2	2	2	1	2	1	2
			**Unbranched uniseriate bicellular**: absent **(1)**, present **(2)**			1	1	1	1	2	1	2	1	2	1	1
			**Unbranched uniseriate multicellular**: absent **(1)**, present **(2)**			1	2	2	2	2	1	1	2	1	1	1
			**Candelabra-like**: absent **(1)**, present **(2)**			2	1	1	1	1	2	1	1	1	1	1
	**Periderm**: absent **(1)**, present **(2)**					2	2	1	1	2	1	1	1	2	1	2
	**Lenticels**: absent **(1)**, present **(2)**					2	2	1	1	2	1	1	1	2	1	2
Ground tissue	*Cortex: Type I **(1)**, Type II **(2)**, Type III **(3)**, Type IV **(4)**, Type V **(5)**, Type VI **(6)**					4	1	2	5	2	6	2	2	3	4	3
	**Pith type**: solid **(1)**, hollow **(2)**					1	1	1	1	1	1	2	1	2	1	1
	**Pith width**: narrow **(1)**, wide **(2)**,					2	2	2	2	2	2	1	2	1	2	2
Vascular system	**Aspect**: dissected cylinder **(1)**, continuous cylinder **(2)**					2	1	1	2	2	2	2	2	2	2	2
	**Xylem vessels**: ring porous **(1)**, diffuse porous **(2)**					2	2	2	2	2	2	2	2	2	2	2
	**Extraxylary fibers**: absent **(1)**, patches **(2)**, complete ring **(3)**					3	2	2	2	2	3	1	3	2	2	2
	** **Horizontal system**: Set I **(1)**, Set II **(2)**, Set III **(3)**, Set IV **(4)**, Set V **(5)**, Set VI **(6)**					1	2	2	1	4	5	1	1	3	1	6
Crystals	**Cortex**: absent **(1)**, druses **(2)**					1	1	1	1	1	2	1	1	1	2	1
	**Pith**: absent **(1)**, druses **(2)**					2	1	1	1	1	2	1	1	1	2	2

(*) Cortex: Type I: Collenchyma patches at corners & 1 row of polyhedral parenchyma; Type II: 3 to 4 rows of collenchyma at corners & 3 rows of polyhedral parenchyma; Type III: 2 to 3 rows of polyhedral parenchyma; Type IV: 5 to 7 rows of polyhedral parenchyma + few stone cells; Type V: 5 rows of collenchyma followed by 5 rows of parenchyma; Type VI: 3 to 5 rows of collenchyma followed by 14 rows of parenchyma

(**) Horizontal system: Set I: Rays uni & biseriate fascicular & interfascicular; Set II: Rays uni & biseriate fascicular only; Set III: Rays uniseriate fascicular & biseriate interfascicular; Set IV: Rays uniseriate at fascicular, fibers only at interfascicular; Set V: Rays uni& biseriate at fascicular, fibers only at interfascicular; Set VI: Rays uniseriate fascicular & interfascicular

**Fig. 2:**
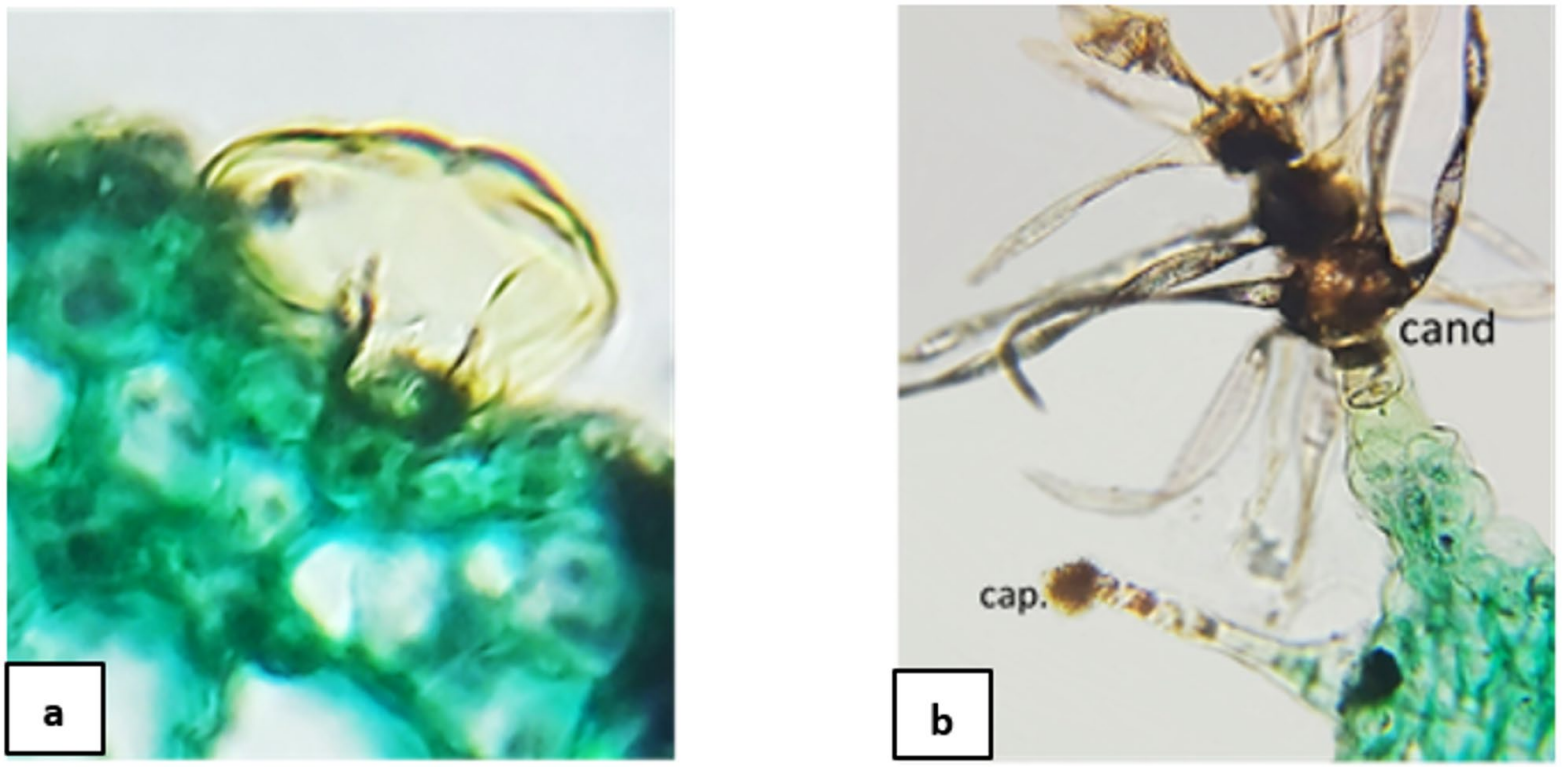
Specific types of trichomes: (**a**) labiaceous trichome structures; (**b**) candelabra and stalked capitate trichomes. cap: capitate trichome, cand: candelabra /dendroid trichome

Ground tissue fluctuates in type and number of rows of its layers. The aspect of the vascular system is diffuse porous, forming a continuous cylinder in all studied taxa except in *M. spicata* and *Ocimum labiatum*, which is dissected. Extraxylary fibers form patches or a continuous ring of sclerenchyma above phloem. In *C. spinosum* and *C. americana* a few stone cells are detected at corners above sclerenchyma fibers. Druses crystals are found in the cortex and pith of both *C. spinosum* and *T. grandis*, and in the pith of *L. camara* and *C. americana* only.

##### Lamina anatomical characteristics (Fig. 1; Table 4)

The lamina is dorsiventral in all studied species, with two to three rows of palisade that are either restricted to the wing, extended to the midrib, or connected ab- and adaxially. The epidermal cells are mostly radial in the midrib and tangential in the wing of the lamina, with thick cuticle in all studied species except *T. vulgaris*. Diverse types of trichomes were identified. Presence of eglandular trichomes (acicular hairs), with high density, was observed in the lamina of all studied taxa except *C. spinosum*, as follows: (a) unbranched unicellular hairs, (b) unbranched uniseriate bicellular hairs, (c) unbranched uniseriate multicellular hairs, and (d) branched candelabra-like trichomes (Fig. 2b), examined in stem and lamina sections of both *T. grandis* and *C. americana* only. Glandular trichomes, with superficial secretory glands, were (a) capitate either sessile or stalked, and (b) peltate: shield-shaped, peltate with multicellular-head, and labiaceous subtype which was noticed abundant on epidermal surface of *M. spicata*, but few in other Lamiaceae members viz. *C. americana*,* O. labiatum*,* S. microphylla*,* T.* vulgaris, whereas, in Verbenaceae it was detected in *L. camara* only (Fig. 2a). In Lamiaceae, crystals were particularly absent in lamina of *M. spicata*, *O. labiatum*, *S. microphylla* and *T. vulgaris*, whereas rhomboid crystals were detected in *Premna odorata*, *T. grandis* and *V. agnus-castus*, and acicular type of crystals were detected only in *P. odorata*. In Verbenaceae, crystals were absent in *A. gratissima*, druses were detected in *L. camara*, and acicular crystals in *C. spinosum.*

**Table 4:** Lamina anatomical characteristics of the studied species (character states and their codes)

Characters & Character States with their Codes						Species										
						1	2	3	4	5	6	7	8	9	10	11
Dermal tissue	**Epidermis**:	At Midrib: tangential **(1)**, radial **(2)**				2	2	2	2	2	2	1	2	2	2	2
		At Wing: tangential **(1)**, radial **(2)**				1	1	1	1	1	1	1	1	1	1	1
	**Cuticle thickness**: thin **(1)**, thick **(2)**					2	2	2	2	2	2	1	2	2	2	2
	**Trichomes**	**Glandular**	**Capitate**	**Stalked**: absent **(1)**, present **(2)**		2	2	2	2	2	2	1	2	1	1	2
				**Sessile**: absent (1), present (2)		2	1	1	1	1	1	2	1	1	1	1
			**Peltate**:	**Shield-shaped**: absent **(1)**, present **(2)**		2	1	1	1	1	1	1	1	1	2	1
				**Multicellular-head**: absent **(1)**, present **(2)**		2	2	2	2	2	2	1	2	1	1	2
				**Labiaceous**: absent **(1)**, seldom **(2)**, abundant **(3)**		2	1	2	1	2	1	2	2	3	1	2
		**Eglandular**	**Unbranched uniseriate unicellular**: absent **(1)**, present **(2)**			2	1	2	1	2	2	2	1	2	1	2
			**Unbranched uniseriate bicellular**: absent **(1)**, present **(2)**			2	1	1	1	2	1	1	1	1	1	1
			**Unbranched uniseriate multicellular**: absent **(1)**, present **(2)**			1	2	2	2	1	1	2	2	1	1	1
			**Candelabra-like**: absent **(1)**, present **(2)**			2	1	1	1	1	2	1	1	1	1	1
*Wing	**Palisade**	**Number of rows**: 1 row (1), 2 rows (2), 3 rows (3)				2	2	2	3	2	2	2	2	2	1	1
		extended **(1)**, restricted to wing **(2)**, connected **(3)**				1	2	2	3	2	2	3	1	3	3	2
		**Intercellular spaces**: present **(1)**, absent **(2)**				1	2	2	2	2	1	2	2	2	1	2
	**Number of rows of spongy layer**: 2 rows **(1)**, 3 rows **(2)**, 4 rows **(3)**, 5 rows **(4)**					2	2	2	2	2	4	1	1	2	3	2
Midrib	**Outline**:	Adaxial: flattened **(1)**, rounded **(2)**, convex **(3)**				3	1	1	3	1	3	1	1	1	3	3
		**Abaxial**: flattened **(1)**, rounded **(2)**, convex **(3)**				2	3	2	2	2	2	3	2	2	2	2
	**Sclerenchyma**: absent **(1)**, patches **(2)**, continuous ring **(3)**, crescent shape above the phloem **(4)**					1	1	1	3	1	3	4	3	1	2	1
Vascular system	**Aspect of siphonostele**: dissected **(1)**, continuous **(2)**					2	2	1	2	2	2	2	2	2	2	2
	**Arrangement**: crescent **(1)**, horseshoe **(2)**, cylinder **(3)**					2	1	1	3	1	3	1	2	1	3	1
	**Accessory (median) bundles adaxially**: absent **(1)**, 1 **(2)**, 2 **(3)**, 3 **(4)**					2	1	1	2	1	4	1	3	1	1	4
Crystals	**Druses**: absent **(1)**, present **(2)**					2	1	1	1	1	2	1	2	1	1	2
	**Acicular**: absent **(1)**, present **(2)**					1	1	1	2	1	1	1	1	1	2	1
	**Rhomboid**: absent **(1)**, present **(2)**					1	1	1	2	1	2	1	2	1	1	1

(*) Wing type: dorsiventral in all studied species

#### GC-MS analysis of EO constituents

The yielded EO, by hydrodistillation, from fresh aerial parts of plants under investigation were differentiated in color into colorless, faint yellow and dull yellow, characterized by their pungent odor to slightly spicy or woody aroma, and yielded 0.48%, 0.07%, 0.006%, 0.15%, 0.2%, 0.3%, 0.02%, 0.12%, 0.003%, 0.27%, 0.08% v/w for *Aloysia gratissima*, *Callicarpa americana*, *Citharexylum spinosum*, *Lantana camara*, *Mentha spicata*, *Ocimum labiatum*, *Premna odorata*, *Salvia microphylla*, *Tectona grandis*, *Thymus vulgaris*, *Vitex agnus-castus* respectively, in relation to the weight of the plant material.

**Fig. 3:**
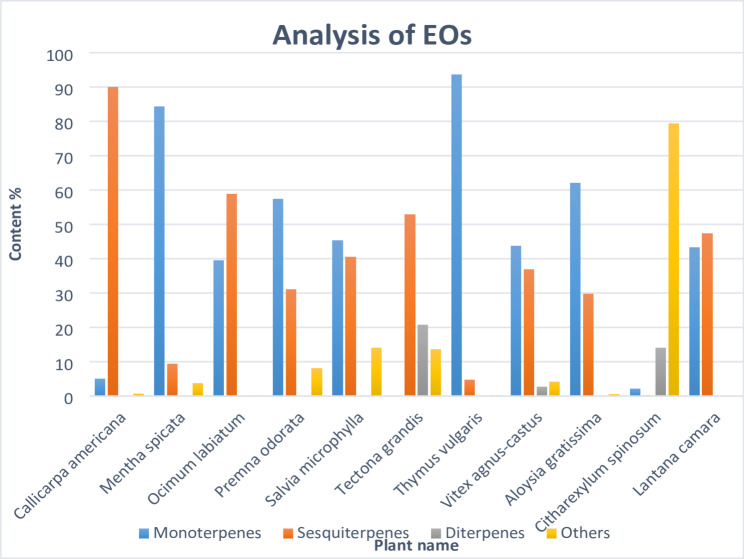
Analytical estimation of essential oils composition in the studied species, showing class variability with their percentage content

From the obtained results of GC-MS analysis (Fig. 3 & S1 -S11; Table 5 & S1), class variability was notable, and the class of monoterpenes was the most abundant class in EO composition of *T*. *vulgaris*, followed by *M. spicata*, *A. gratissima*, *P. odorata*, *S. microphylla*, *V. agnus-castus*, *L. camara* and *O. labiatum*, with percentage order 93.7%, 84.44% 62.66%, 57.47%, 45.33%, 43.75%, 43.32% and 39.6%. On the other hand, monoterpenes inclusion was lower in *T. grandis*, *C. spinosum* and *C. americana*, with percentages 0%, 2.19% and 5.08%, respectively.

**Table 5:** The total number of compounds and their chemical classes for all the studied species

Class		Species										
		1	2	3	4	5	6	7	8	9	10	11
Monoterpenes		8	17	11	12	9	0	19	16	16	4	12
Sesquiterpenes		24	11	18	12	7	11	7	13	28	2	10
Diterpenes		0	1	0	0	0	2	0	2	0	6	0
Others		1	8	1	4	0	5	1	6	0	19	1

Among the monoterpenes; *p*-Cymene, D-Sylvestrene, Eucalyptol, *γ*-Terpinene, Piperitone oxide, Isobornyl acetate, Thymol and Carvone oxide were represented by the highest percentages in several taxa belong to Lamiaceae family (above 15%), particularly, *p*-Cymene was detected in *T. vulgaris* with high percentage (22.07%), and in other taxa of Lamiaceae, it was detected with low percentages in *S. microphylla* (1.13%), *O. labiatum* (0.41%) and *V. agnus-castus* (0.1%), but it was absent in all taxa of Verbenaceae. *α*-Phellandrene was also a monoterpene detected in several species of Lamiaceae, but absent in all species of Verbenaceae. *α-*Pinene, *β*-Myrcene, and *α*-Terpineol were commonly detected in most of the studied species that belong to both Lamiaceae and Verbenaceae families, showing significant differences, as declared by the data in Table S1. Moreover, Sabinene was the highest monoterpene in both *A. gratissima* and *L. camara* that belong to the Verbenaceae family.

Sesquiterpenes were the prevailing volatile oil constituents in *C. americana* (90.01%) and *O. labiatum* (58.98%), and considerable in *L. camara* (47.34%), *T. grandis* (45.31%), *S. microphylla* (40.64%), *V. agnus-castus* (38.25%), *P. odorata* (31.08%) and *A. gratissima* (29.84%). Germacrene D, *cis-β*-Farnesene, Valencene, Caryophyllene oxide, and Juniper camphor were the highest detected sesquiterpenes. Diterpenes were either undetected or found in trace amounts in all taxa, except in *C. spinosum* and *T. grandis*; dominated by Phytol. On the other hand, other constituents and volatile substances are relatively neglectable, except in the case of *C. spinosum *were major and dominated by *β-*Phenylethyl benzoate and Phenylethyl salicylate. Caryophyllene was the most common sesquiterpene, as it is detected in all studied species except in *S. microphylla* and *T. grandis*.

#### Cytotoxic Activity of Essential Oils (EOs)

Essential-oil-bearing plants that produced high yield of EO were testedin-vitro against hepatocellular carcinoma (HepG 2) cell lines, and the obtained results were evidenced by the IC_50_ values being 34.5, 36.7, 48.3, and 58.9 µg/mL for *Ocimum labiatum*, *Lantana camara*, *Salvia microphylla* and *Mentha spicata*, respectively (Table 6). The essential oil of *Thymus vulgaris* showed mild cytotoxicity (43.9% at 100ppm), meanwhile, the essential oils of *Vitex agnus-castus*, *Callicarpa americana* and *Aloysia gratissima* showed minor cytotoxic activity, 13.5%, 12.8% and 10.6% at 100ppm, respectively.

**Table 6:** In-vitro bioassay on human tumor cell line (HepG 2)

Plant name	IC_50_ (µg/mL)	% of cytotoxicity
*Ocimum labiatum*	34.5 ± 0.7	100% at 100ppm
*Lantana camara*	36.7 ± 0.3	100% at 100ppm
*Salvia microphylla*	48.3 ± 0.8	100% at 100ppm
*Mentha spicata*	58.9 ± 1.4	85.5% at 100ppm
DMSO	-	1% at 100ppm
Doxorubicin (Dox)	21.6 ± 1.2	95%
Negative control	-	0%

IC_50_: Inhibition concentration of the sample that causes the death of 50% of cells in 48 h

## Discussion

### Morphological characters

Significant morpho-anatomical differences between Lamiaceae and Verbenaceae have been inspected, revealing that Lamiaceae species are trees, shrubs, subshrubs or herbs developing woody to herbaceous stems, bear radially symmetrical (actinomorphic) or bilaterally symmetrical (zygomorphic) flowers aggregated in verticillasters, later form schizocarpic fruits with four nutlets or drupes, while Verbenaceae species are woody trees, shrubs or subshrubs, bear flowers in panicles vary in color and form (raceme or spike), and this result agrees with investigations by previous studies [1, 12, 13]. As informed by Huxley [12], the observations revealed that all studied species are perennials, monoecious with bisexual flowers arranged in panicles or verticillasters of variable forms. The morphological character of opposite decussate leaves with symmetric lamina bases is shared by both families. The presence of eglandular trichomes (acicular hairs) is observed in stem and lamina of all studied taxa except *Citharexylum spinosum*. This agrees with investigations by many authors reporting that eglandular trichomes are observed in higher density than glandular ones [17, 27]. Meanwhile, glandular trichomes, with superficial secretory glands, including capitate and peltate ones, e.g. Labiaceous subtype which is specialized to Lamiaceae plants, for producing essential oils and other compounds, are noticed to be abundant on the epidermal surface in *Mentha spicata*, but few in other members of Lamiaceae and *Lantana camara*, as investigated by El-Kashoury et al. [38].

### Phytochemical study

The yielded EO is directly related to terpenoid secretion that has been found to be restricted to presence of glandular secretory trichomes on plant organs, such glandular structures represent the primary sites of secondary metabolites biosynthesis, secretion and accumulation, and generally consist of either simple subcutaneous glands or trichomes, particularly labiaceous type, from where essential oils are produced [18, 38–40].

Lamiaceae is one of the most important families producing essential oils that can be synthesized by all plant organs due to the wide distribution of oil glands (labiaceous subtype of glandular trichomes) that are intimately correlated to species belonging to Lamiaceae [41, 42]. Labiaceous trichomes are frequently found in some Verbenaceae plants such as *L. camara* and *Aloysia gratissima* as noticed in the current study (Tables 3 and 4).

From the obtained results of GC-MS, class variability was notable, and monoterpenes was the main class in the EO composition of the examined species possessed labiaceous trichome structures in *Thymus vulgaris*, *A. gratissima*, *Salvia microphylla*, *Premna odorata*, *L. camara*, *M. spicata*, *Vitex agnus-castus* and *Ocimum labiatum*. This result, compared to data by other authors as Karpiński [4], indicates that monoterpenes are particularly common in EO of species belong to Lamiaceae, and detected frequently in other studied taxa that belong to Verbenaceae showing great variability in percentage, taking into account that metabolic variation in the EO composition is attributed to many factors influencing the chemical constituents of the essential oils [43–45]. This means that the frequency and type of trichomes (glandular trichomes) are directly related to the EO yield and composition.

High density of glandular trichomes observed in *M. spicata* and *T. vulgaris* where monoterpenoid-rich essential oils yielded high values; 0.2% and 0.27% respectively. In *C. spinosum*, only a few shield subtype of peltate glandular trichomes were detected, and consequently, the yielded essential oil was little (0.006% v/w), and rich in non-terpenoid constituents. Also, in *T. grandis*, which possessed few glandular trichomes, the yield of essential oil was very little (0.006% v/w). Meanwhile, peltate and capitate trichomes were frequently observed in the remaining species, from where mono- and sesquiterpenes were obtained in considerable amounts (Fig. 3). Thus, the results of the current study supported the hypothesis that the high density of glandular trichomes is positively correlated with the high yield of EO rich in terpenoids.

However, to date, there has been no extensive detailed report on diversity of trichomes (types and secreted compounds) in both Lamiaceae and Verbenaceae, additionally, the relation between the density of trichomes and volatile terpenoid contents in the aerial parts of their species has not yet been determined, Chen et al. [46] reviewed the correlation between the glandular trichomes and EO production and pointed to the crucial role of the peltate and capitate trichomes in *T. vulgaris*, specified that peltate ones are the primary sites for biosynthesis of Thymol monoterpene. *Chen* et al. and Adeniran et al. [46, 47]. also highlighted the peltate and capitate trichomes in different *Ocimum* spp. where secretion of monoterpenoids, particularly, Eugenol and Linalool, takes place.

Among monoterpenes, the oxygenated form of Carvone (Carvone oxide) is the highest compound exclusively detected in *M. spicata* (38.72%). However, the investigation by Weecharangsan et al. [48]. reported that *M. spicata* contained Limonene and Carvone as major compounds, D-Limonene has a low percentage in the current study (1.88%). Thymol is another monoterpene detected in *M. spicata* (0.72%) and dominated the essential oil composition of *T. vulgaris* (40.16%), and according to many studies, Thymol is a major EO active component in *T. vulgaris* and other *Thymus spp* [49–52]. Additionally, Sabinene monoterpene dominated the essential oils of both *A. gratissima* and *L. camara*, which are Verbenaceae members, with percentages 51.02% and 15.40% respectively, as shown in Table S1, and this agreed with the results by Solar et al. and Ramírez [53, 54], although *C. spinosum* was the lowest in monoterpene content among all the studied species.

Sesquiterpenes were reported in Lamiaceae by many authors, but not so in Verbenaceae [55]. In the current study, sesquiterpenes were generally prevailing volatile oil constituents in *C. americana*, *O. labiatum*, and *Tectona grandis* which are species in the Lamiaceae family. Additionally, *P. odorata*, *S. microphylla* and *V. agnus-castus* possess considerable concentrations of sesquiterpene compounds. On the other hand, the Verbenaceae members; *L. camara* and *A. gratissima*, also possess considerable concentrations of sesquiterpene compounds. The most common sesquiterpenes identified in the vast majority of the studied species were Caryophyllene, followed by its oxygenated form (Caryophyllene oxide), Humulene and τ-Cadinol [23].

Such essential-oil-bearing plants are medicinally valuable due to their secondary metabolites in essential oils [39]. Although there are no previous studies on the cytotoxic activity of EOs extracted from the species under investigation in this work on hepatocellular carcinoma (HepG 2), few studies reported that EO of other related taxa that belong to Lamiaceae and Verbenaceae showed a significant effect on cell viability of HepG 2 [56, 57]. This consolidates the obtained result of this study in which EO of most species belong to Lamiaceae showed significant activity evidenced by the IC_50_ (Table 6). These findings ascertain the better replacement of naturally occurring bio-active and preservative chemical compounds to synthetic additives for pharmaceutical and nutraceutical industries.

Overall, this work is conducted for the first time, gathering all these species that belong to both Lamiaceae and Verbenaceae families, correlating their morphological and anatomical features alongside their EOs constituents, in addition to the biological activity of their yielded oils. Moreover, notable variations were obtained among the EOs compositions of the studied species, with attempts to highlight the compounds that had the highest concentrations, and the others that were mostly common among many taxa, particularly the major compounds that belong to the monoterpene and sesquiterpene classes.

## Conclusion

This work attempts to examine species from both the Lamiaceae and Verbenaceae families in order to correlate their macro- and micromorphological features of the stem and lamina with their essential oil (EO) constituents and their biological activity. Significant morpho-anatomical differences reported among Lamiaceae and Verbenaceae members were useful for species discrimination, particularly, the anatomical traits such as types of trichomes, occurrence of crystals, and the shape of midrib, represent important taxonomic markers. GC-MS results revealed notable class variability in the EO composition. Monoterpenes were commonly found in the EO of Lamiaceae taxa, and frequently detected in Verbenaceae taxa, although metabolic variation leads to great variability in their percentages, as they were identified as the main class in the EO composition of *Thymus vulgaris* (93.7%), *Mentha spicata* (84.44%) and *Aloysia gratissima* (62.66%). Whereas sesquiterpenes were generally prevailing volatile oil constituents in other species in the Lamiaceae family, such as *Callicarpa americana* (90.01%), *Ocimum labiatum* (58.98%) and *Tectona grandis* (45.31%). Sesquiterpenes were also present in considerable concentrations in various members of Lamiaceae and Verbenaceae. As the glandular trichomes varied in occurrence (present/ absent), types (peltate/ capitate) and density (abundant/ few/ seldom), accordingly, the yield and composition of the obtained EOs also varied. Biological study revealed the significant effect of EO of most species belong to Lamiaceae on cell viability, ensuring that EO could serve as a promising anticancer agent that contributes to naturally occurring drugs, and these findings indicate that the differences in bioactivity were related to the differences of chemical composition. For the obtained results to be ascertained, further studies on other members of the two families are required.

## Supplementary Information

Supplementary Material 1: Table S1: Volatile Constituents of the Aerial parts of the Studied Taxa. Figures S1-11: showing GC–MS chromatogram of each plant.

## Data Availability

All data generated or analyzed during this study are included inside the manuscript and/or its supplementary information files.

## Abbreviations

EO: Essential oil
GC-MS: Gas chromatography - mass spectrometry
HepG 2: Human liver cancer cell line
IC_50_: Inhibition concentration of the sample which causes the death of 50% of cells in 48 h
LM: Light microscope
