# Supplementary material for: Integrating morphological and phytochemical studies on some selected taxa of Lamiaceae Lindl. and Verbenaceae Juss
Source: BMC Plant Biol. 2026 May 20;26:895. doi: 10.1186/s12870-026-08910-2 (PMC13188656; doi:10.1186/s12870-026-08910-2)
Supplement: Supplementary file 1 — Supplementary Material 1: Table S1: Volatile Constituents of the Aerial parts of the Studied Taxa. Figures S1-11: showing GC–MS chromatogram of each plant. [file 12870_2026_8910_MOESM1_ESM.pdf]

Table S1. Volatile constituents of the aerial parts of the studied taxa.

No	RT	Compound Name	MF	RI		Content %										
				Calculated	Reported	1	2	3	4	5	6	7	8	9	10	11
1.	6.999	*α*-Phellandrene	$C_{10}H_{16}$	926	971	0.11	-	3.07	-	-	-	1.03	0.32	-	-	-
2.	7.001	Origanene	$C_{10}H_{16}$	926	927	-	-	-	-	-	-	-	-	1.13	-	-
3.	7.183	*α*-Pinene	$C_{10}H_{16}$	932	932	0.63	1.50	-	13.95	8.08	-	0.89	0.64	2.54	-	4.48
4.	7.610	Camphene	$C_{10}H_{16}$	946	946	-	-	-	0.13	7.91	-	0.93	-	0.11	-	1.36
5.	8.377	Sabinene	$C_{10}H_{16}$	972	972	-	1.22	-	5.07	-	-	-	3.93	51.02	-	15.40
6.	8.455	*β*-Pinene	$C_{10}H_{16}$	975	975	3.30	1.68	0.40	-	4.24	-	0.22	0.64	-	-	3.14
7.	8.602	1-Octen-3-ol	$C_8H_{16}O$	980	979	0.70	-	0.19	7.42	-	-	0.34	1.61	-	-	-
8.	8.804	3-Octanone	$C_8H_{16}O$	987	985	-	-	-	-	-	-	-	0.28	-	-	-
9.	8.921	*β*-Myrcene	$C_{10}H_{16}$	991	991	-	1.35	5.39	1.76	-	-	1.12	0.77	0.39	-	1.20
10.	9.091	3-Octanol	$C_8H_{18}O$	997	996	-	0.94	-	0.32	-	-	-	0.20	-	-	-
11.	9.399	3-Carene	$C_{10}H_{16}$	1007	1007	-	-	-	-	-	-	-	-	-	-	1.53
12.	9.588	4-Carene	$C_{10}H_{16}$	1013	1014	-	-	-	1.32	-	-	-	-	-	-	-
13.	9.682	*α*-Terpinene	$C_{10}H_{16}$	1016	1016	0.11	-	-	-	-	-	1.01	1.43	0.48	-	-
14.	9.933	*o*-Cymene	$C_{10}H_{14}$	1024	1027	-	-	-	-	-	-	-	-	0.59	-	-
15.	9.942	*p*-Cymene	$C_{10}H_{14}$	1024	1024	-	-	0.41	-	1.13	-	22.07	0.10	-	-	-
16.	10.057	D-Limonene	$C_{10}H_{16}$	1028	1028	-	1.88	-	-	-	-	-	-	-	-	-
17.	10.057	D-Sylvestrene	$C_{10}H_{16}$	1028	1028	0.34	-	-	31.09	-	-	-	-	2.30	-	-
18.	10.161	Eucalyptol	$C_{10}H_{18}O$	1031	1031	-	4.34	6.89	-	14.01	-	2.22	27.41	-	-	10.04
19.	10.264	*trans*-*β*-Ocimene	$C_{10}H_{16}$	1035	1039	-	-	10.65	-	-	-	-	-	-	-	-
20.	10.688	*β*-Ocimene	$C_{10}H_{16}$	1049	1050	-	-	2.38	0.20	-	-	-	0.22	0.65	-	-
21.	11.004	*γ*-Terpinene	$C_{10}H_{16}$	1059	1062	0.17	-	-	0.72	1.08	-	15.18	3.07	0.86	-	-
22.	11.264	*cis*-4-Thujanol	$C_{10}H_{18}O$	1067	1064	-	-	-	-	-	-	0.64	-	1.62	-	0.87
23.	11.875	Clorius	$C_8H_8O_2$	1096	1106	-	-	-	-	-	-	-	-	-	4.33	-
24.	11.923	Isoterpinolene	$C_{10}H_{16}$	1088	1088	-	-	-	-	-	-	-	0.61	0.27	-	-
25.	12.285	Nonanol	$C_9H_{20}O$	1100	1101	-	-	-	-	-	3.43	-	-	-	-	-
26.	12.289	Linalool	$C_{10}H_{18}O$	1100	1100	0.28	-	0.72	2.28	-	-	1.73	0.51	-	-	-
27.	12.290	Isopentyl 2-methylbutanoate	$C_{10}H_{20}O_2$	1100	1101	-	0.44	-	-	-	-	-	-	-	-	-
28.	12.450	Valeric acid	$C_{10}H_{20}O_2$	1105	1106	-	0.25	-	-	-	-	-	0.23	-	-	-
29.	12.947	*trans*-2-Menthenol	$C_{10}H_{18}O$	1122	1120	-	-	-	-	-	-	-	0.23	0.17	-	-
30.	13.031	3-Octanol, acetate	$C_{10}H_{20}O_2$	1124	1124	-	0.30	-	-	-	-	-	-	-	-	-

No	RT	Compound Name	MF	RI		Content %										
				Calculated	Reported	1	2	3	4	5	6	7	8	9	10	11
31.	13.397	Cyclohexanone,2-(1-methylethylidene)-	$C_9H_{14}O$	1136	1151	-	0.27	-	-	-	-	-	-	-	-	-
32.	13.504	*d*-Camphor	$C_{10}H_{16}O$	1140	1141	-	-	0.61	-	4.99	-	0.84	-	-	-	1.63
33.	13.511	*cis*-2-Menthenol	$C_{10}H_{18}O$	1140	1139	-	-	-	-	-	-	-	0.15	-	-	-
34.	13.695	(1R)-cis-Verbenol	$C_{10}H_{16}O$	1146	1142	-	-	-	-	-	-	-	-	0.09	-	-
35.	13.839	*p*-Vinylanisole	$C_9H_{10}O$	1151	1151.6	-	-	-	0.13	-	-	-	-	-	-	-
36.	14.074	*α*-Acetoxytoluene	$C_9H_{10}O_2$	1167	1170	-	-	-	-	-	-	-	-	-	0.63	-
37.	14.335	Borneol	$C_{10}H_{18}O$	1167	1173	-	-	8.70	-	2.62	-	2.05	-	0.16	-	1.18
38.	14.369	*δ*-Terpineol	$C_{10}H_{18}O$	1168	1167	-	-	-	-	-	-	-	0.44	-	-	-
39.	14.371	*α*-Terpineol	$C_{10}H_{18}O$	1192	1192	-	0.29	0.38	-	-	-	0.21	3.28	-	0.96	1.11
40.	14.677	4-Carvomenthenol	$C_{10}H_{18}O$	1178	1178	0.14	0.16	-	-	-	-	-	-	-	-	1.38
41.	14.946	*p*-cymenol	$C_{10}H_{14}O$	1187	1186	-	0.14	-	-	-	-	-	-	-	-	-
42.	14.981	Methyl salicylate	$C_8H_8O_3$	1196	1198	-	-	-	-	-	-	-	-	-	1.82	-
43.	15.273	Myrtenol	$C_{10}H_{16}O$	1198	1198	-	-	-	-	-	-	-	-	0.28	-	-
44.	16.092	*cis*-Myrtanol	$C_{10}H_{18}O$	1226	1234	-	-	-	0.21	-	-	-	-	-	-	-
45.	16.265	*O*-Methylthymol	$C_{11}H_{16}O$	1232	1233	-	-	-	-	-	-	1.16	-	-	-	-
46.	16.354	Cuminal	$C_{10}H_{12}O$	1244	1246	-	-	-	-	-	-	-	-	-	0.29	-
47.	16.536	Methyl carvacrol	$C_{11}H_{16}O$	1241	1241	-	-	-	-	-	-	0.68	-	-	-	-
48.	16.834	*β*-Phenethyl acetate	$C_{10}H_{12}O_2$	1261	1260	-	-	-	-	-	-	-	-	-	5.60	-
49.	16.868	*cis*-Geraniol	$C_{10}H_{18}O$	1252	1251	-	-	-	0.18	-	-	-	-	-	-	-
50.	17.100	Piperitone oxide	$C_{10}H_{16}O_2$	1260	1253	-	30.83	-	-	-	-	-	-	-	-	-
51.	17.415	2-Cyclohexen-1-one, 2-hydroxy-6-methyl-3-(1-methylethyl)-	$C_{10}H_{16}O_2$	1271	1274.1	-	0.37	-	-	-	-	-	-	-	-	-
52.	17.534	Isopiperitenone	$C_{10}H_{14}O$	1275	1272	-	0.18	-	-	-	-	-	-	-	-	-
53.	17.745	Bornyl acetate	$C_{12}H_{20}O_2$	1282	1277	-	-	-	-	-	-	0.13	-	-	-	-
54.	17.767	Isobornyl acetate	$C_{12}H_{20}O_2$	1283	1288	-	-	-	-	15.28	-	-	-	-	-	-
55.	17.958	*o*-Thymol	$C_{10}H_{14}O$	1300	1300	-	-	-	-	-	-	1.43	-	-	0.40	-
56.	17.999	Dihydroedulan II	$C_{13}H_{22}O$	1291	1296	-	-	-	0.29	-	-	-	-	-	-	-
57.	18.195	Thymol	$C_{10}H_{14}O$	1298	1296	-	0.72	-	-	-	-	40.16	-	-	-	-
58.	18.326	Diosphenol	$C_{10}H_{16}O_2$	1302	1305	-	0.57	-	-	-	-	-	-	-	-	-
59.	18.578	Nonyl acetate	$C_{11}H_{22}O_2$	1311	1315	-	0.24	-	-	-	-	-	-	-	-	-
60.	19.359	δ-EIemene	$C_{15}H_{24}$	1340	1340	-	-	-	-	-	-	-	-	0.59	-	-

No	RT	Compound Name	MF	RI		Content %										
				Calculated	Reported	1	2	3	4	5	6	7	8	9	10	11
61.	19.485	Hydroxycineyl acetate	$C_{12}H_{20}O_3$	1344	1345.5	-	-	-	-	-	-	-	0.25	-	-	-
62.	19.520	Piperitenone	$C_{10}H_{14}O$	1345	1343	-	0.34	-	-	-	-	-	-	-	-	-
63.	19.630	*β*-Citronellyl acetate	$C_{12}H_{22}O_2$	1349	1354	-	-	-	0.56	-	-	-	-	-	-	-
64.	20.318	Carvone oxide	$C_{10}H_{14}O_2$	1374	1369	-	38.72	-	-	-	-	-	-	-	-	-
65.	20.456	Copaene	$C_{15}H_{24}$	1379	1380	-	0.52	0.70	1.41	-	-	-	-	3.34	-	-
66.	20.559	*α*-Bourbonene	$C_{15}H_{24}$	1383	1384	-	-	0.68	-	-	-	-	-	-	-	-
67.	20.706	*β*-Bourbonene	$C_{15}H_{24}$	1388	1388	-	-	-	-	-	-	-	-	0.26	-	-
68.	20.882	*β*-Elemen	$C_{15}H_{24}$	1394	1394	-	-	0.68	1.20	-	-	-	-	0.68	-	-
69.	21.177	Cinerolone	$C_{10}H_{14}O_2$	1405	1426	-	1.21	-	-	-	-	-	-	-	-	-
70.	21.386	*α*-Gurjenene	$C_{15}H_{24}$	1413	1411	-	-	-	-	-	-	-	0.31	0.68	-	-
71.	21.512	Aromandendrene	$C_{15}H_{24}$	1460	1460	-	-	0.44	-	-	4.93	-	-	-	-	-
72.	21.541	*cis*-*β*-Farnesene	$C_{15}H_{24}$	1419	1438	-	-	-	-	22.83	-	-	-	-	-	-
73.	21.664	Caryophyllene	$C_{15}H_{24}$	1424	1424	0.59	3.39	8.42	13.48	-	-	2.64	9.71	3.64	0.13	13.91
74.	21.765	*cis*-*β*-Copaene	$C_{15}H_{24}O$	1428	1428	-	-	0.19	-	-	-	-	-	-	-	-
75.	21.993	*γ*-Elemene	$C_{15}H_{24}$	1437	1437	-	-	0.36	-	-	-	-	-	0.59	-	-
76.	22.225	*β*-Sesquiphellandrene	$C_{15}H_{24}$	1445	1446	-	-	-	-	-	-	-	0.36	-	-	-
77.	22.243	Geranyl acetone	$C_{13}H_{22}O$	1456	1456	-	-	-	-	-	-	-	-	-	0.54	-
78.	22.563	Humulene	$C_{15}H_{24}$	1458	1457	5.12	-	2.28	3.51	0.67	-	-	-	0.56	-	7.01
79.	22.397	2,6,10-trimethyltridecane	$C_{16}H_{34}$	1462	1461	-	-	-	-	-	-	-	-	-	1.01	-
80.	22.553	*trans*-*β*-Famesene	$C_{15}H_{24}$	1458	1458	-	0.74	-	-	-	-	-	5.33	-	-	-
81.	22.763	Alloaromadendrene	$C_{15}H_{24}$	1466	1466	-	-	0.22	-	-	1.71	-	1.84	0.53	-	-
82.	23.005	*α*-Amorphene	$C_{15}H_{24}$	1475	1475	-	-	-	-	-	1.09	-	-	-	-	-
83.	23.158	γ-Muurolene	$C_{15}H_{24}$	1481	1481	1.13	-	-	-	-	-	-	0.17	0.60	-	-
84.	23.298	Germacrene D	$C_{15}H_{24}$	1487	1485	-	2.14	18.95	1.23	-	-	0.24	-	5.02	-	-
85.	23.388	4a,8-Dimethyl-2-(prop-1-en-2-yl)-1,2,3,4,4a,5,6,7-octahydronaphthalene	$C_{15}H_{24}$	1490	1491.8	11.98	-	-	-	-	-	-	-	-	-	-
86.	23.401	10,11-Epoxycalamenene	$C_{15}H_{20}O$	1490	1485	-	-	-	0.59	-	-	-	-	-	-	-
87.	23.465	*β*-Eudesmene	$C_{15}H_{24}$	1493	1492	5.85	-	-	-	-	-	-	-	-	-	-
88.	23.495	*α*-Guaiene	$C_{15}H_{24}$	1494	1490	-	-	-	-	-	-	-	-	0.18	-	-
89.	23.522	Benzyl tiglate	$C_{12}H_{14}O_2$	1506	1498	-	-	-	-	-	-	-	-	-	0.21	-
90.	23.632	Cubebol	$C_{15}H_{26}O$	1499	1495	-	-	-	-	-	-	-	-	0.54	-	0.70
91.	23.642	Valencene	$C_{15}H_{24}$	1500	1496	19.40	-	-	-	-	-	-	-	-	-	-

No	RT	Compound Name	MF	RI		Content %										
				Calculated	Reported	1	2	3	4	5	6	7	8	9	10	11
92.	23.695	Bicyclogermacrene	$C_{15}H_{24}$	1502	1501	-	-	-	-	-	-	-	11.06	0.40	-	1.04
93.	23.763	*α*-Muurolene	$C_{15}H_{24}$	1505	1505	0.59	-	-	-	-	-	-	-	0.52	-	-
94.	23.944	(R)-lavandulyl (R)-2-methylbutanoate	$C_{16}H_{28}O_2$	1512	1504	-	0.15	-	-	-	-	-	-	-	-	-
95.	24.124	γ-Cadinene	$C_{15}H_{24}$	1519	1515	0.65	0.32	-	-	-	-	-	-	-	-	-
96.	24.169	*4-epi*-cubebol	$C_{15}H_{26}O$	1521	1619	0.19	-	3.10	-	-	-	-	-	1.58	-	-
97.	24.225	1,1,4,5,6-Pentamethyl-2,3-dihydro-1H-indene	$C_{14}H_{20}$	1523	1522.6	-	-	-	-	-	-	-	0.13	-	-	-
98.	24.265	*α*-Panasinsanene	$C_{15}H_{24}$	1525	1527	9.78	-	-	-	-	-	-	-	-	-	-
99. -	24.349	*δ*-Cadinene	$C_{15}H_{24}$	1528	1528	1.72	0.25	2.09	4.19	-	1.40	-	0.34	1.87	-	-
100.	24.466	*trans*-*β*-Ionone	$C_{13}H_{20}O$	1480	1503	-	-	-	-	-	3.18	-	-	-	-	-
101.	24.575	Cadine-1,4-diene	$C_{15}H_{24}$	1537	1539	0.11	-	-	-	-	-	-	-	-	-	-
102.	24.674	γ-selinene	$C_{15}H_{24}$	1541	1544	0.91	-	-	-	-	-	-	-	-	-	-
103.	24.839	Selina-3,7(11)-diene	$C_{15}H_{24}$	1548	1547	0.68	-	-	-	-	-	-	-	-	-	-
104.	24.999	Elemol	$C_{15}H_{26}O$	1554	1552	-	-	-	-	-	-	-	-	0.12	-	-
105.	25.155	*β-Bisabolene*	$C_{15}H_{24}$	1561	1547	-	-	-	0.18	-	-	-	-	-	-	-
106.	25.186	*trans*-Nerolidol	$C_{15}H_{26}O$	1562	1562	-	-	-	-	-	-	-	-	-	-	12.48
107.	25.242	*β*-Germacrene	$C_{15}H_{24}$	1564	1562	1.05	0.15	14.92	-	-	-	-	-	2.06	-	-
108.	25.395	Davanone	$C_{15}H_{24}O_2$	1570	1568	-	-	-	-	-	-	-	-	-	-	0.68
109.	25.507	Palustrol	$C_{15}H_{26}O$	1575	1571	-	-	-	-	-	-	-	0.87	-	-	-
110.	25.753	Spathulenol	$C_{15}H_{24}O$	1585	1582	-	-	3.22	-	-	-	-	2.01	-	-	1.74
111.	25.769	*β*-Copaen-4α-ol	$C_{15}H_{24}O$	1585	1585	-	-	-	-	-	-	-	-	0.42	-	-
112.	25.899	Caryophyllene oxide	$C_{15}H_{24}O$	1591	1592	0.50	0.14	-	4.31	2.84	18.28	0.46	-	1.14	-	5.97
113.	25.903	Viridiflorol	$C_{15}H_{24}O$	1591	1590	-	-	1.11	-	-	-	-	2.73	-	-	-
114.	26.127	Lauryl acetate	$C_{14}H_{28}O_2$	1609	1610	-	-	-	-	-	-	-	-	-	0.21	-
115.	26.278	Humulene epoxide I	$C_{15}H_{24}O$	1606	1596	0.62	-	-	0.11	-	-	-	-	-	-	-
116.	26.541	Humulene-1,2-epoxide	$C_{15}H_{24}O$	1617	1610	2.76	-	-	0.55	-	4.30	-	-	-	-	2.71
117.	26.650	*Epi*-cubenol	$C_{15}H_{26}O$	1622	1622	-	0.13	0.39	-	-	-	-	-	-	-	-
118.	26.906	8-epi-γ-Eudesmol	$C_{15}H_{26}O$	1633	1626	-	-	-	-	-	-	0.33	-	-	-	-
119.	26.971	Isospathulenol	$C_{15}H_{24}O$	1636	1638	-	-	-	-	-	-	-	-	0.85	-	-
120.	27.043	11,11-Dimethyl-4,8-dimethylenebicyclo[7.2.0]undecan-3-ol	$C_{15}H_{24}O$	1639	1645.9	-	-	-	-	-	2.63	-	-	-	-	-

No	RT	Compound Name	MF	RI		Content %										
				Calculated	Reported	1	2	3	4	5	6	7	8	9	10	11
121.	27.086	Humulenol-II	$C_{15}H_{24}O$	1641	1632	1.87	-	-	-	-	-	-	-	-	-	-
122.	27.272	τ-Cadinol	$C_{15}H_{26}O$	1649	1649	-	1.50	0.99	0.32	-	1.66	-	3.18	0.51	0.17	1.10
123.	27.365	*β*-Eudesmol	$C_{15}H_{26}O$	1653	1654	-	-	0.24	-	3.79	-	0.44	-	-	-	-
124.	27.384	*α*-Muurolol	$C_{15}H_{26}O$	1653	1654	1.25	-	-	-	-	-	-	-	0.28	-	-
125.	27.436	*α*-Eudesmol	$C_{15}H_{26}O$	1656	1657	-	-	-	-	6.02	-	-	-	-	-	-
126.	27.439	γ-Eudesmol	$C_{15}H_{26}O$	1656	1646	-	-	-	-	3.11	-	0.66	-	-	-	-
127.	27.587	τ-Muurolol	$C_{15}H_{26}O$	1662	1654	-	-	-	-	-	-	-	0.34	0.28	-	-
128.	27.644	Neointermedeol	$C_{15}H_{26}O$	1664	1662	2.83	-	-	-	-	-	-	-	-	-	-
129.	27.769	Juniper camphor	$C_{15}H_{26}O$	1670	1675	16.87	-	-	-	-	-	-	-	-	-	-
130.	27.856	Isoaromadendrene epoxide	$C_{15}H_{24}O$	1673	1612	-	-	-	-	-	4.42	-	-	-	-	-
131.	28.308	Cubenol	$C_{15}H_{26}O$	1693	1676	-	-	-	-	1.38	-	-	-	-	-	-
132.	28.324	Isogermacrene D	$C_{15}H_{24}$	1693	1515	-	-	-	-	-	-	0.18	-	-	-	-
133.	28.348	(1R,7S,E)-7-Isopropyl-4,10-dimethylenecyclodec-5-enol	$C_{15}H_{24}O$	1694	1694.5	0.20	-	-	-	-	1.71	-	-	0.17	-	-
134.	28.470	Eudesma-4(15),7-dien- *β*-ol	$C_{15}H_{24}O$	1700	1690	-	-	-	-	-	-	-	-	0.13	-	-
135.	28.669	Pentadecanal	$C_{15}H_{30}O$	1708	1700	-	-	-	-	-	2.05	-	-	-	-	-
136.	29.892	Benzyl Benzoate	$C_{14}H_{12}O_2$	1774	1775	-	-	-	-	-	-	-	-	-	12.00	-
137.	30.665	1-Tetradecyl acetate	$C_{16}H_{32}O_2$	1809	1811	-	-	-	-	-	-	-	-	-	0.40	-
138.	30.892	Dehydrofukinone	$C_{15}H_{22}O$	1799	1813	-	0.19	-	-	-	-	-	-	2.30	-	-
139.	31.190	Acetate, (2,4a,5,8a-tetramethyl-1,2,3,4,4a,7,8,8a-octahydro-1-naphthalenyl) ester	$C_{16}H_{26}O_2$	1814	1709	3.25	-	-	-	-	-	-	-	-	-	-
140.	31.458	2-Pentadecanone, 6,10,14-trimethyl	$C_{18}H_{36}O$	1847	1847	-	-	-	-	-	-	-	-	-	2.73	-
141.	31.532	Phytone	$C_{18}H_{36}O$	1832	1835	-	-	-	-	-	7.55	-	-	-	-	-
142.	31.874	*β*-Phenylethyl benzoate	$C_{15}H_{14}O_2$	1867	1860	-	-	-	-	-	-	-	-	-	22.72	-
143.	32.165	Benzyl salicylate	$C_{14}H_{12}O_3$	1881	1877.4	-	-	-	-	-	-	-	-	-	7.89	-
144.	33.135	Hexadecanoic acid, methyl ester	$C_{17}H_{34}O_2$	1928	1927	-	-	-	-	-	-	-	-	-	0.76	-
145.	34.061	Phenylethyl salicylate	$C_{15}H_{14}O_3$	1973	1987	-	-	-	-	-	-	-	-	-	18.11	-

No	RT	Compound Name	MF	RI		Content %										
				Calculated	Reported	1	2	3	4	5	6	7	8	9	10	11
146.	34.140	Palmitic acid	$C_{16}H_{32}O_2$	1964	1964	-	0.20	-	-	-	-	-	-	-	0.27	-
147.	34.954	Manool oxide	$C_{20}H_{34}O$	2005	2002	-	0.15	-	-	-	-	-	1.24	-	-	-
148.	35.245	Geranyl linalool	$C_{20}H_{34}O$	2033	2034	-	-	-	-	-	-	-	-	-	2.00	-
149.	35.657	Hexadecanoic acid, trimethylsilyl ester	$C_{20}H_{42}O_2Si$	2041	2047	-	-	-	-	-	-	-	-	-	-	0.90
150.	36.470	Sclareol	$C_{20}H_{36}O_2$	2196	2198	-	-	-	-	-	-	-	0.26	-	0.29	-
151.	36.489	Heneicosane	$C_{21}H_{44}$	2099	2109	-	-	-	-	-	-	-	-	-	0.60	-
152.	36.569	Methyl elaidate	$C_{19}H_{36}O_2$	2103	2085	-	-	-	-	-	-	-	-	-	0.27	-
153.	36.920	Phytol	$C_{20}H_{40}O$	2107	2107	-	-	-	-	-	20.77	-	-	-	4.10	-
154.	38.743	Phytol acetate	$C_{22}H_{42}O$	2220	2223	-	-	-	-	-	-	-	-	-	4.64	-
155.	40.359	4,9,13,17-Tetramethyl-4,8,12,16-octadecatetraenal	$C_{22}H_{36}O$	2312	2337	-	-	-	-	-	-	-	-	-	0.48	-
156.	43.566	Tetracosane	$C_{24}H_{50}$	2487	2400	-	-	-	-	-	3.11	-	-	-	0.15	-
157.	44.464	Di-n-octyl phthalate	$C_{24}H_{38}O_4$	2544	2545	-	-	-	-	-	2.78	-	-	-	-	-
158.	46.351	Phenethyl palmitate	$C_{24}H_{40}O_2$	2684	2651	-	-	-	-	-	-	-	-	-	0.27	-
159.	46.661	Heptacosane	$C_{27}H_{56}$	2686	2705	-	-	-	-	-	2.32	-	-	-	-	-
160.	48.503	Squalene	$C_{30}H_{50}$	2831	2833	-	-	-	-	-	-	-	-	-	1.87	-
**Monoterpenes**						5.08	84.44	39.6	57.47	45.33	-	93.7	43.75	62.66	2.19	43.32
**Sesquiterpenes**						90.01	9.47	58.98	31.08	40.64	45.31	4.77	38.25	29.84	0.3	47.34
**Diterpenes**						-	0.15	-	-	-	28.32	-	1.5	-	14.03	-
**Others**						0.70	3.85	0.19	8.16	-	13.69	0.34	2.7	-	79.33	0.90
**Total identified components**						95.79	97.91	98.77	96.71	99.98	87.32	98.81	86.2	92.5	95.85	91.56

The names of these compounds are in order of their elution from the Rtx-5MS column.
Identification is based on comparison of the compounds' mass spectral data (MS) and retention indices (RI) with those of NIST Mass Spectral Library (October 2025) [33].

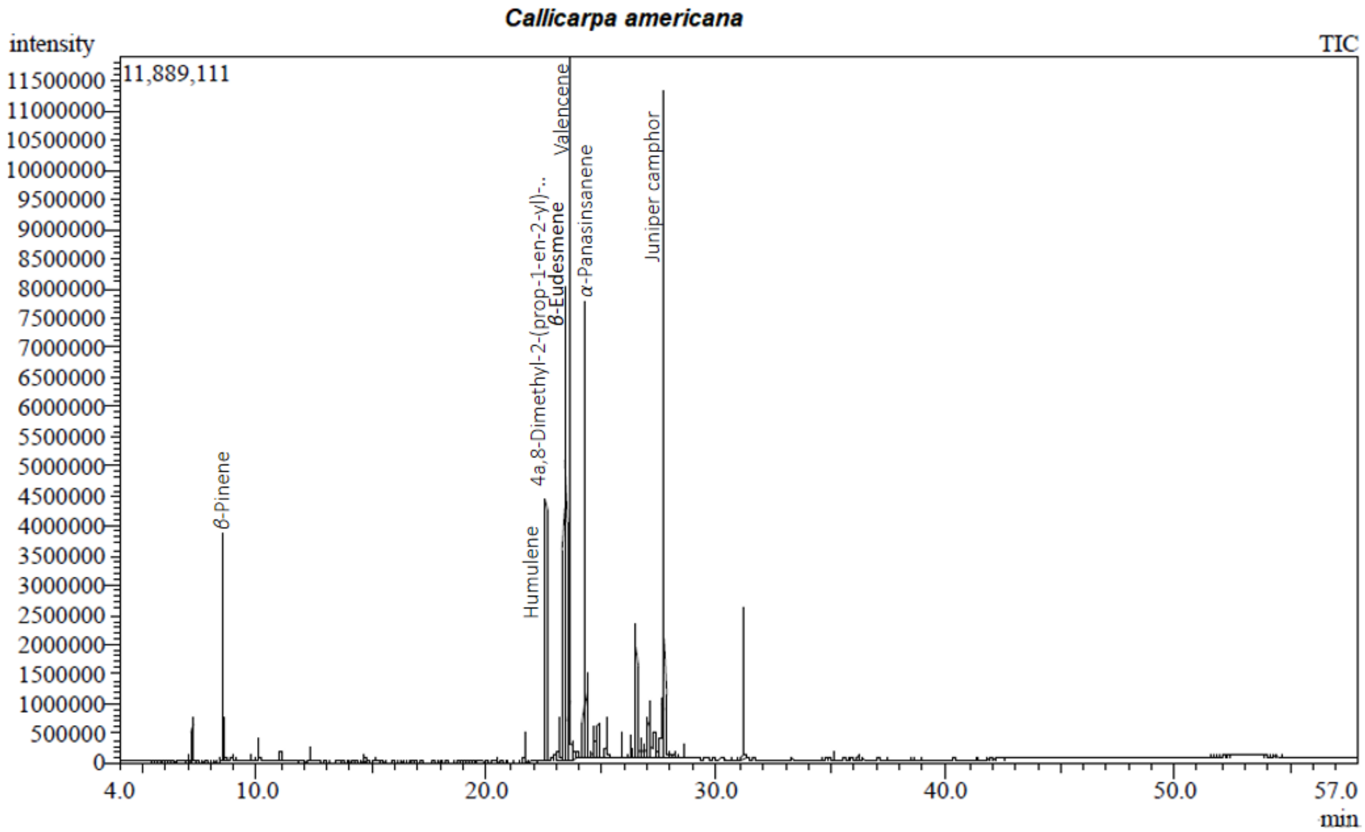

Figure S1: GC-MS chromatogram of volatile constituents in *Callicarpa americana*

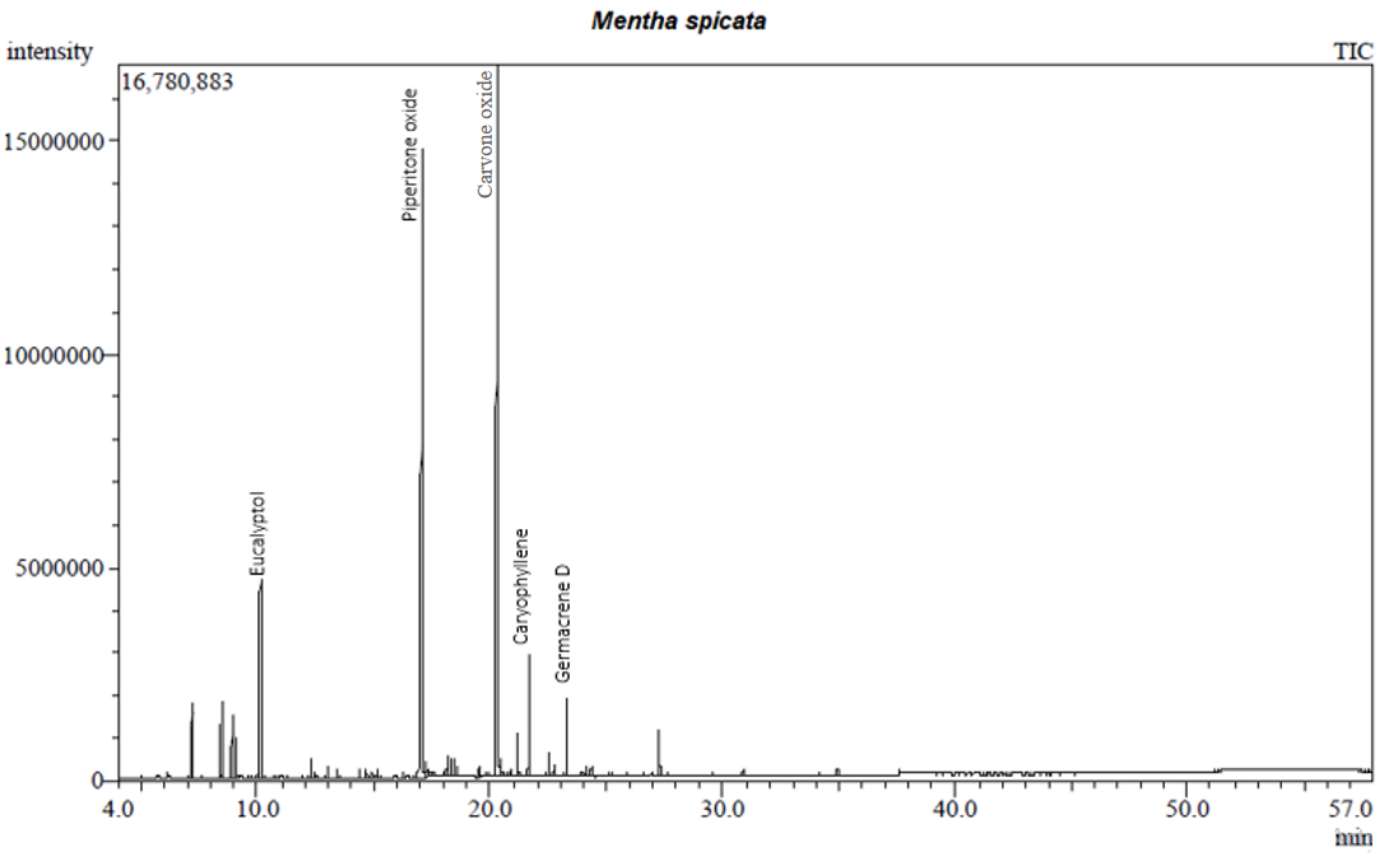

Figure S2: GC-MS chromatogram of volatile constituents in *Mentha spicata*

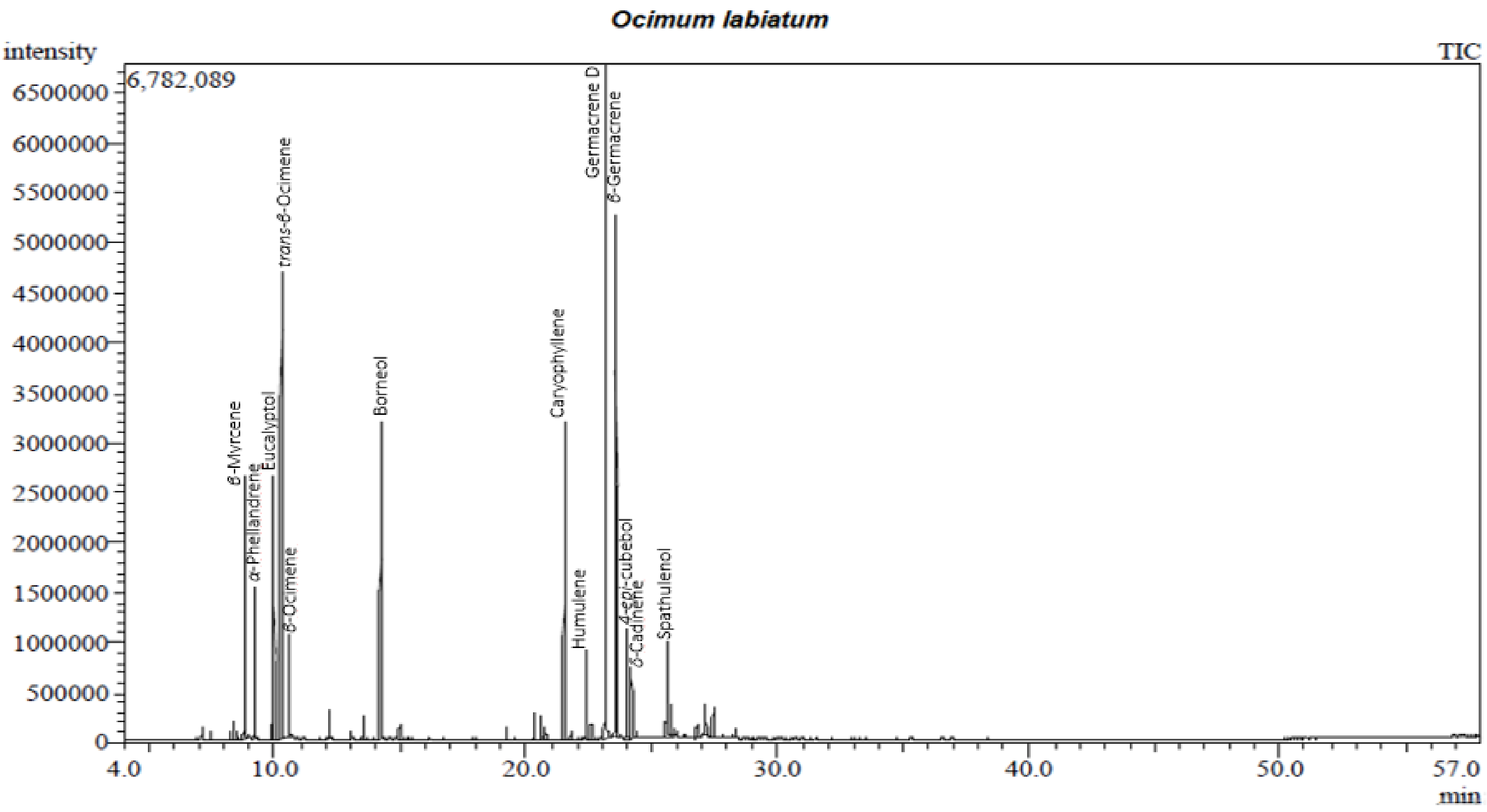

Figure S3: GC-MS chromatogram of volatile constituents in *Ocimum labiatum*

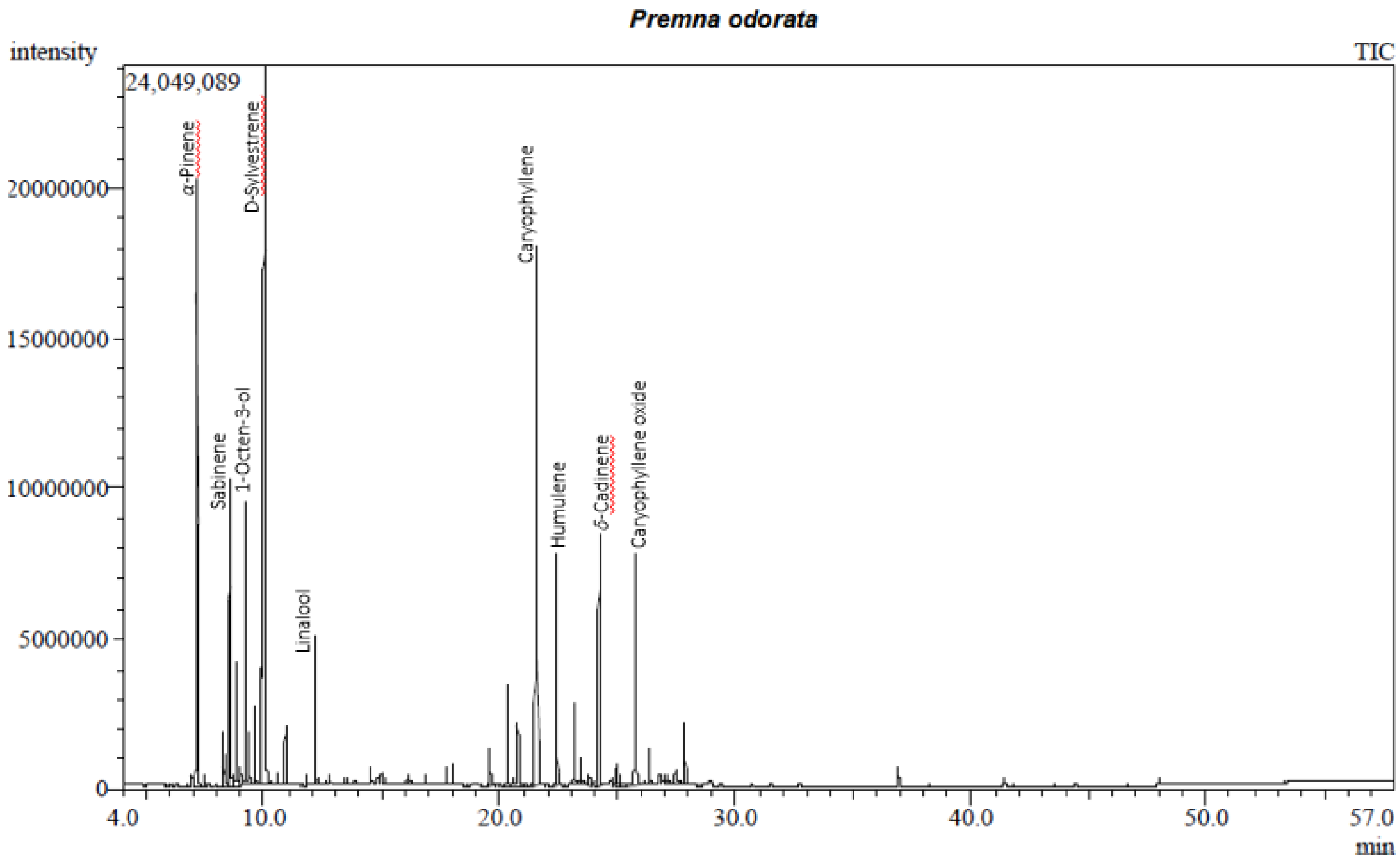

Figure S4: GC-MS chromatogram of volatile constituents in *Premna odorata*

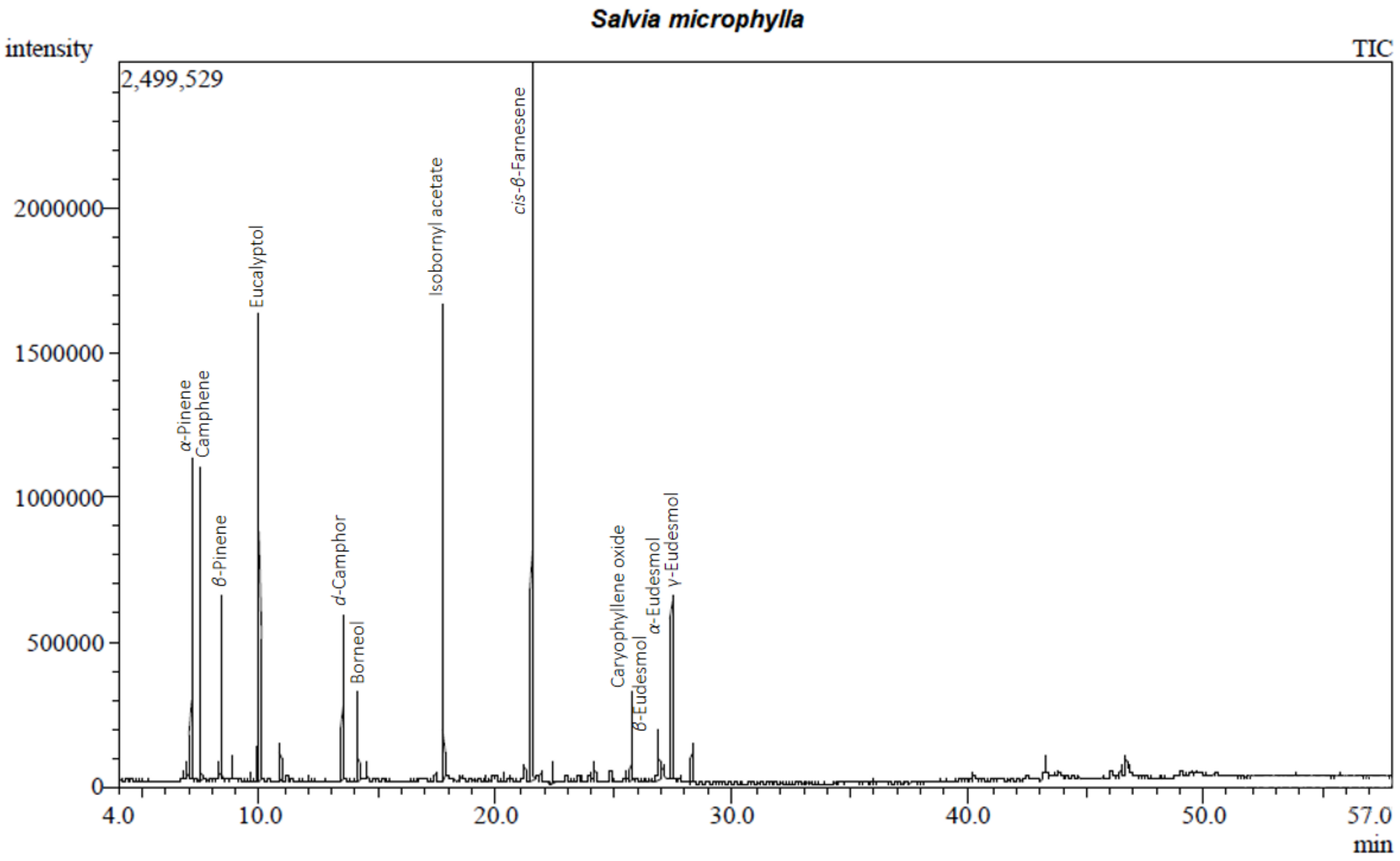

Figure S5: GC-MS chromatogram of volatile constituents in *Salvia microphylla*

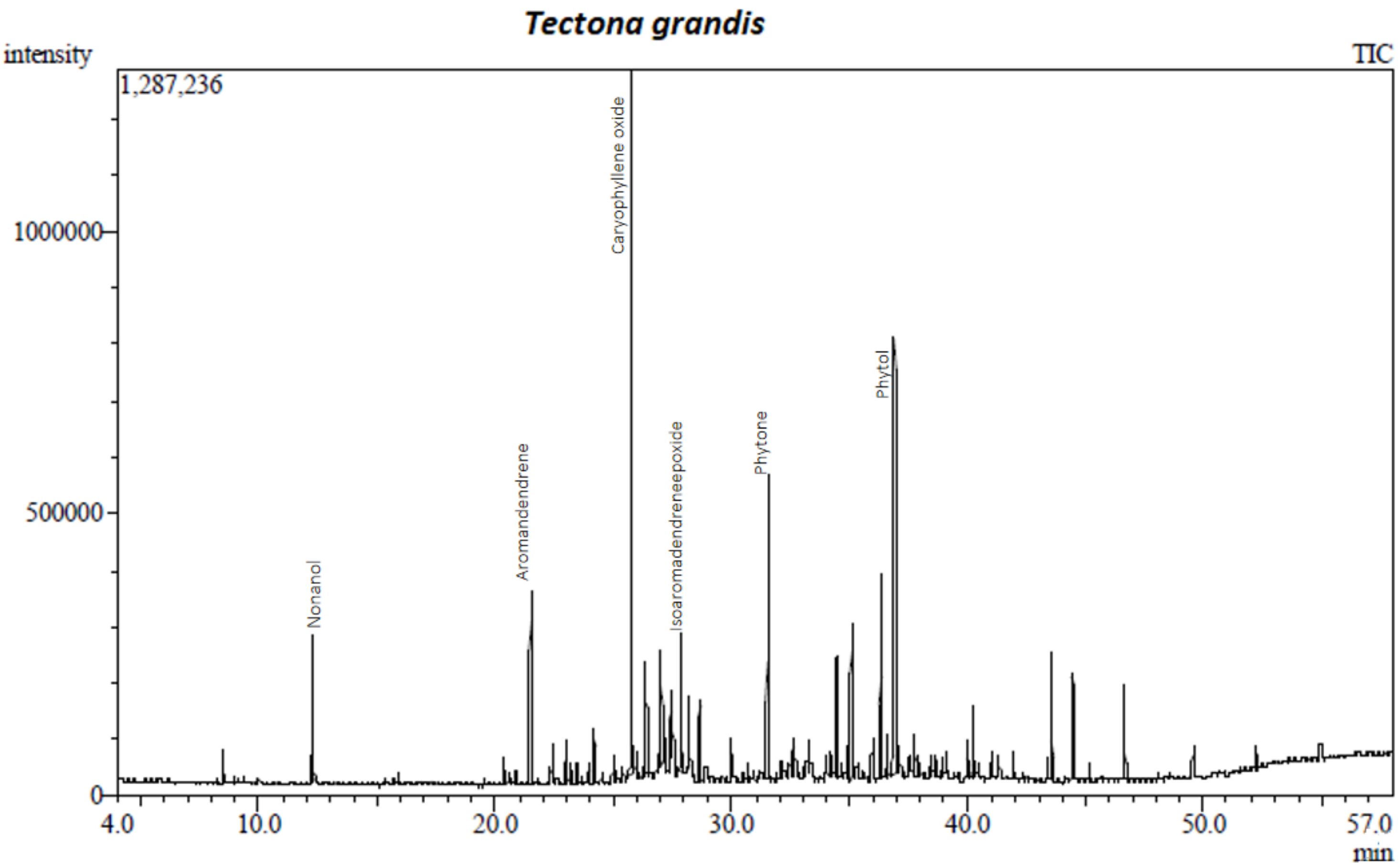

Figure S6: GC-MS chromatogram of volatile constituents in *Tectona grandis.*

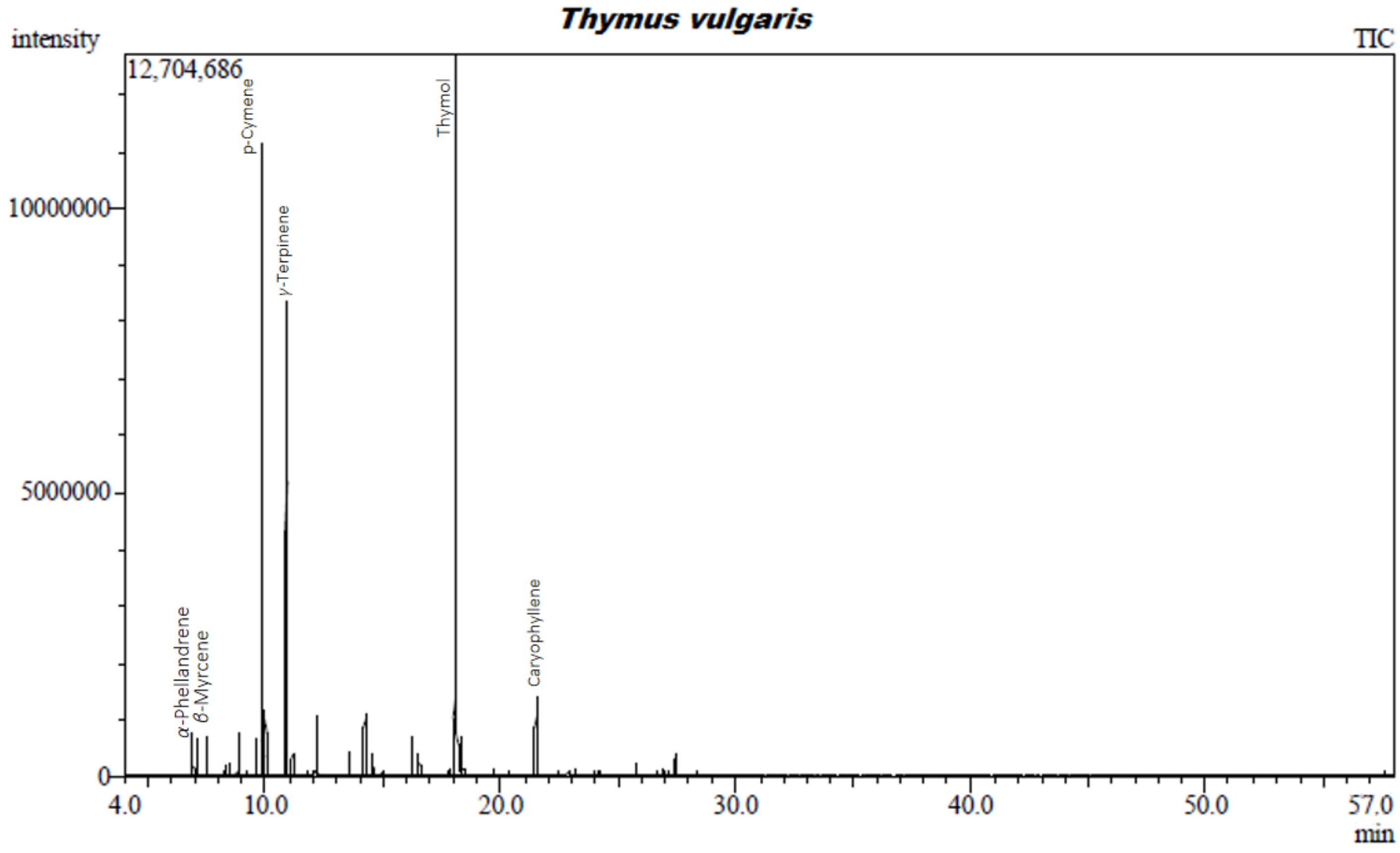

Figure S6: GC-MS chromatogram of volatile constituents in *Thymus vulgaris.*

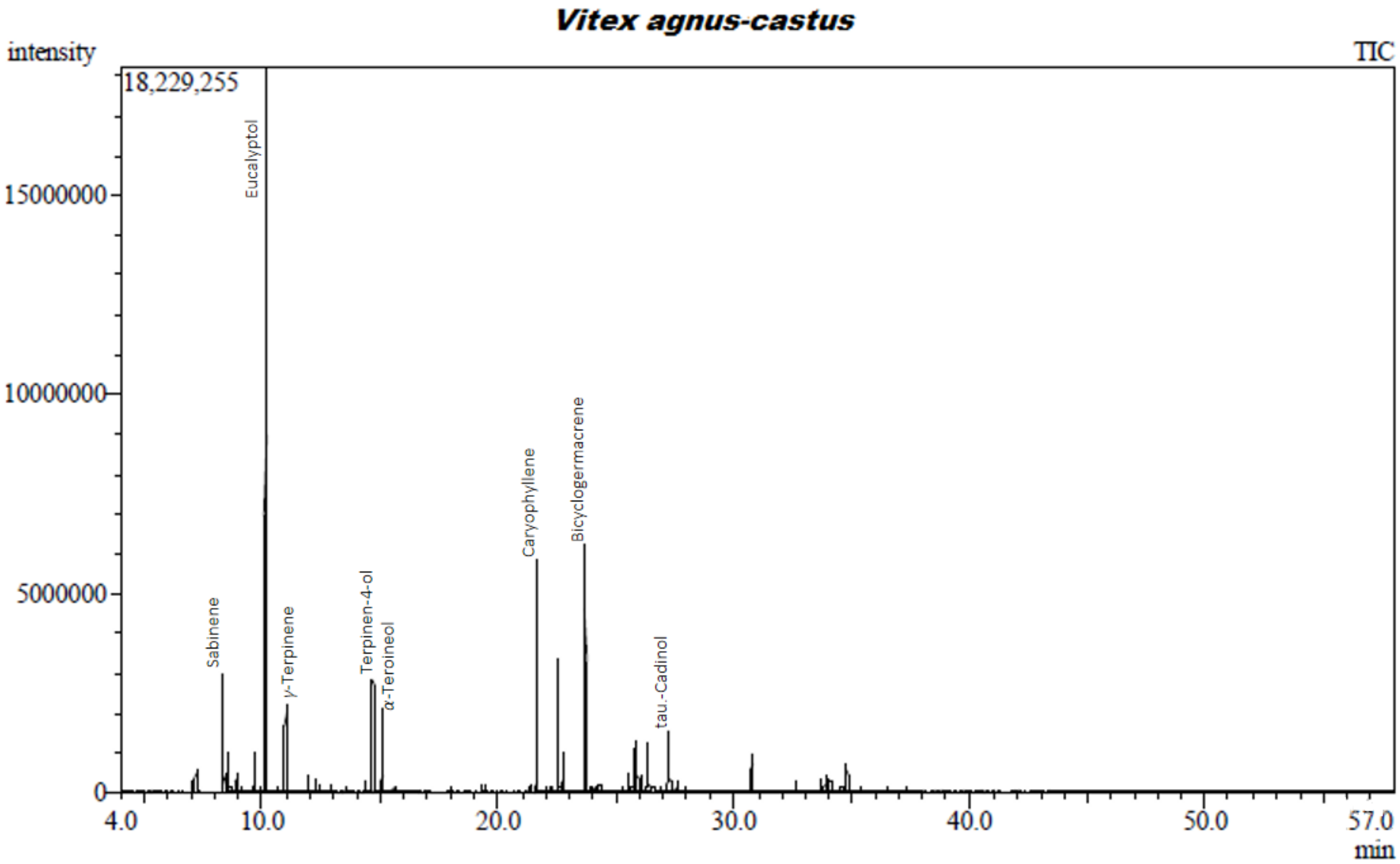

Figure S8: GC-MS chromatogram of volatile constituents in *Vitex agnus-castus.*

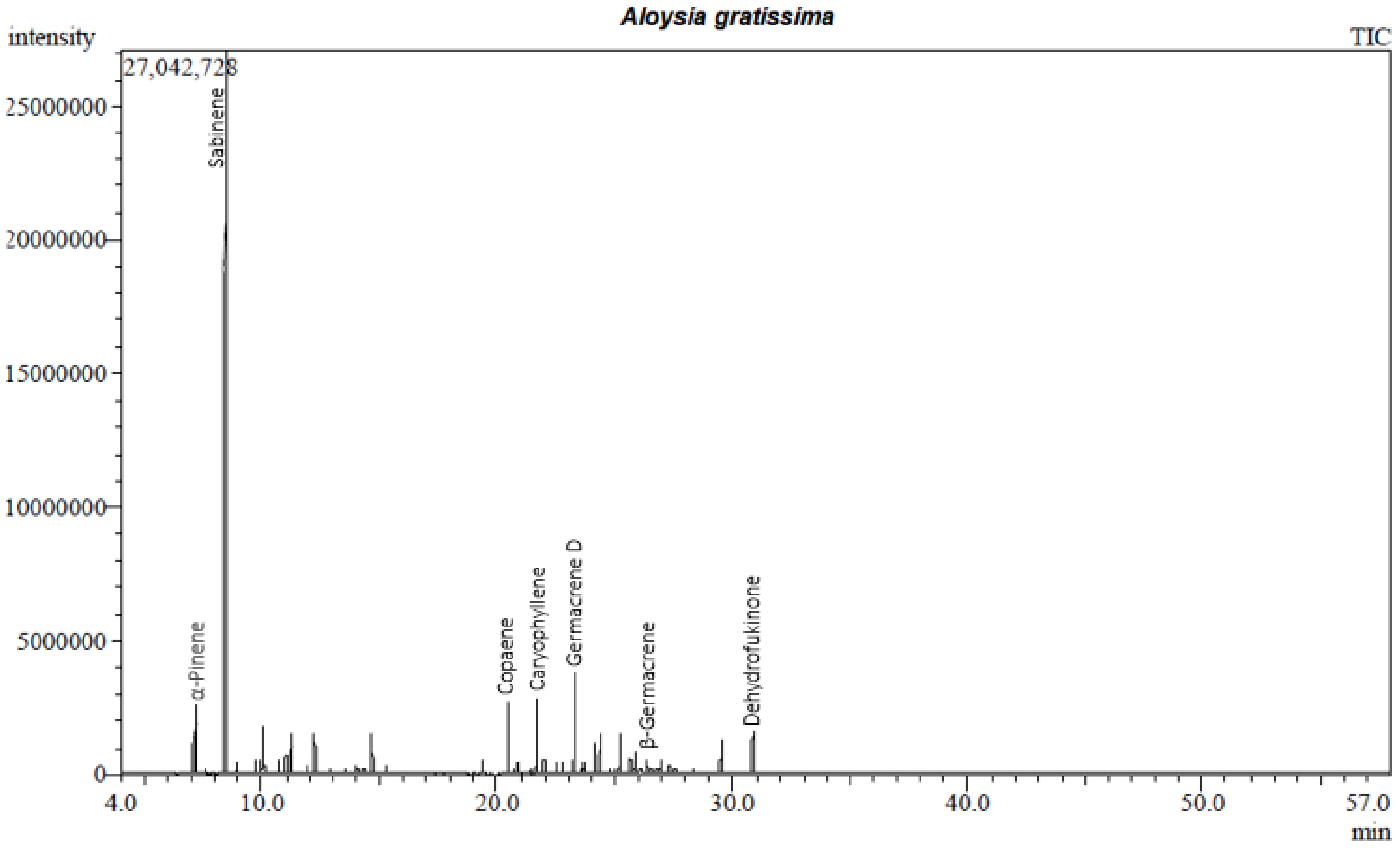

Figure S9: GC-MS chromatogram of volatile constituents in *Aloysia gratissima*

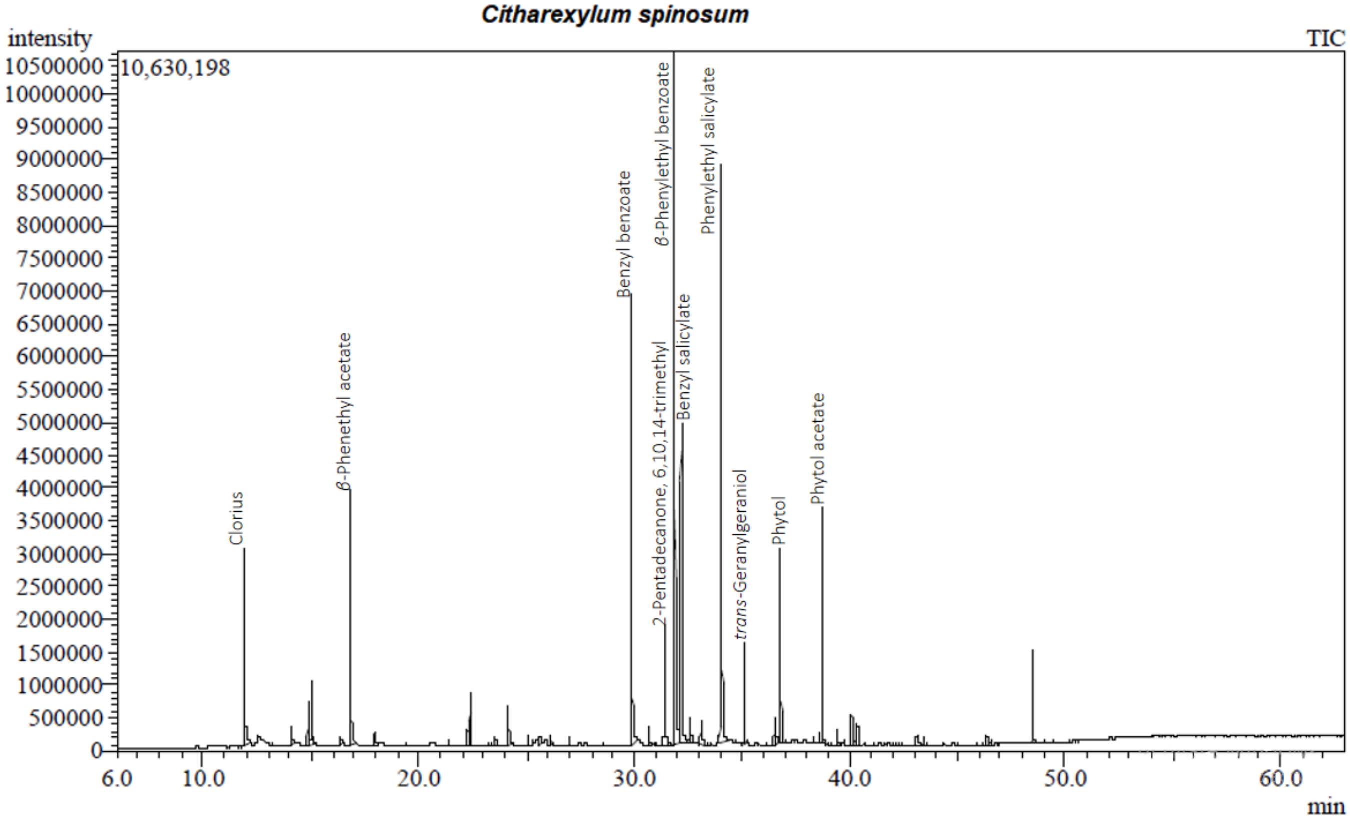

Figure S10: GC-MS chromatogram of volatile constituents in *Citharexylum spinosum*

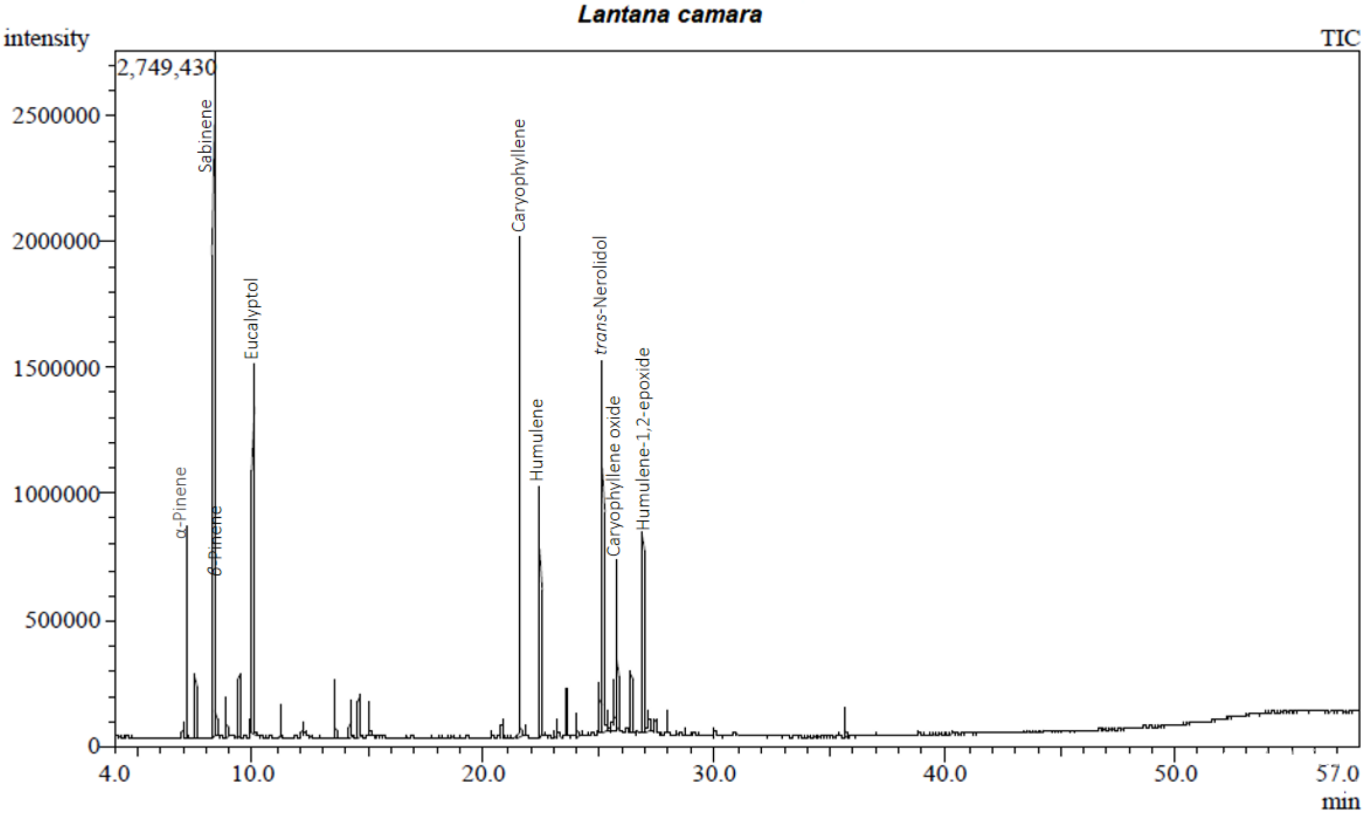

Figure S11: GC-MS chromatogram of volatile constituents in *Lantana camara*
